# Sex Differences in the Efficacy of Glucagon‐Like Peptide‐1 Receptor Agonists for Weight Reduction: A Systematic Review and Meta‐Analysis

**DOI:** 10.1111/1753-0407.70063

**Published:** 2025-03-05

**Authors:** Yucheng Yang, Liyun He, Shumeng Han, Na Yang, Yiwen Liu, Xuechen Wang, Ziyi Li, Fan Ping, Lingling Xu, Wei Li, Huabing Zhang, Yuxiu Li

**Affiliations:** ^1^ Department of Endocrinology, Key Laboratory of Endocrinology of National Health Commission, Translation Medicine Center, Peking Union Medical College Hospital Chinese Academy of Medical Sciences & Peking Union Medical College Beijing China; ^2^ Diabetes Research Center of Chinese Academy of Medical Sciences Beijing China

## Abstract

**Aim:**

To verify sex differences of GLP‐1RAs for weight reduction.

**Methods:**

We searched RCTs reporting weight change by sex from PubMed, Web of Science, Embase, Cochrane Library, and ClinicalTrials registries. Meta‐regression was performed to evaluate the association between weight reduction and sex differences. Subgroup analyses were stratified by individual GLP‐1RA medications, dose, treatment duration, indication, type of control, background treatment, and baseline weight. The study protocol was registered (CRD42023480167).

**Results:**

Fourteen studies covering dulaglutide, exenatide, liraglutide, semaglutide, and retatrutide were included in this study. The meta‐analysis showed that females lost more weight than males (MD 1.04 kg [95% CIs 0.70–1.38]; MD 1.69% [95% CI 0.78–2.61]). The pooled results of GLP‐1RAs indicated similar results (MD 0.88 kg [95% CIs 0.67–1.09]). Meta‐regression illustrated that substantial weight reduction was significantly relevant to greater gender differences (*β* = −0.19 [95% CIs −0.29 to −0.09]). Subgroup analysis demonstrated that indications for weight reduction increased the gender difference in weight reduction (MD 4.21 kg [95% CIs 1.75–6.67]). Background treatment, dose, duration of treatment, baseline weight, and type of control had no subgroup differences in the sex difference in weight reduction of GLP‐1RAs. Dulaglutide (MD 0.88 kg [95% CIs 0.63–1.12]) and semaglutide (MD 1.04 kg [95% CIs 0.45–1.63]) showed statistically significant differences in weight reduction between males and females. No gender difference was observed in the exenatide subgroup analysis.

**Conclusions:**

Females lost more weight than males when treated with GLP‐1RAs for weight reduction. The sex difference in weight reduction became more pronounced as the degree of weight reduction increased. Indications for obesity could magnify this sex difference.

## Graphical abstract

1. GLP‐1 RAs would lead to more weight reduction in females than in males.
2. The sex difference in weight reduction became more pronounced as the degree of weight reduction increased.

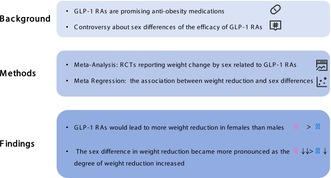

**Summary**

- GLP‐1 RAs would lead to more weight reduction in females than males.
- The sex difference in weight reduction became more pronounced as the degree of weight reduction increased.
- Indications for obesity could magnify the sex difference in weight reduction of GLP‐1 RAs.

## 1 Introduction

The obesity epidemic is accelerating globally and is closely associated with an increased risk for a wide range of diseases, including diabetes mellitus (DM) and cardiovascular disease (CVD) [1]. Glucagon‐like peptide‐1 (GLP‐1) receptor agonists (GLP‐1 RAs) exert weight reduction properties, making them promising antiobesity medications. As such, determining the extent to which this weight reduction effect is influenced by specific factors would positively affect their clinical application(s), thereby enabling a more precise estimation of the weight loss outcomes attributed to GLP‐1 RAs.

Whether sex significantly influences the efficacy of GLP‐1 RAs for weight reduction remains controversial. Some studies have suggested that the extent of weight reduction induced by GLP‐1 RAs may be affected by sex [2]. Other studies have indicated that males and females experienced similar weight reductions during treatment with GLP‐1 RAs [3, 4]. However, these studies were limited by sample sizes and study designs. Furthermore, labels for GLP‐1 RAs did not indicate any discernible sex differences in weight reduction outcomes [5, 6, 7, 8]. As such, a comprehensive analysis of this sex difference for GLP‐1 RAs is required.

Thus, the objective of the present systematic review and meta‐analysis was to identify potential sex differences in the efficacy of GLP‐1 RAs for weight loss, complemented by additional analyses to investigate factors that may influence this sex difference.

## 2 Materials and Methods

This systematic review and meta‐analysis was performed in accordance with the Preferred Reporting Items for Systematic Reviews and Meta‐Analysis (i.e., “PRISMA”) guidelines [9]. The study protocol was prospectively registered with PROSPERO (ID: CRD42023480167).

### 2.1 Search Strategy and Study Selection

The PubMed, Web of Science, Embase, Cochrane Library, and ClinicalTrials.gov databases were searched for relevant studies published from inception to August 31, 2023 (last search, September 10, 2023). Two independent reviewers (Y.Y. and S.H.) performed the searches using predesigned strategies (Table S1). The reference lists of selected studies and previously published systematic reviews were also reviewed. Two investigators (Y.Y. and S.H.) independently reviewed the databases and screened the titles, abstracts, and full‐text articles to select the studies. If necessary, consensus was achieved with the assistance of other team members.

### 2.2 Inclusion and Exclusion Criteria

All human studies investigating GLP‐1 RAs that reported weight reduction results stratified according to sex were included. The inclusion criterion was randomized controlled trials (RCTs) lasting > 12 weeks in adults with or without type 2 DM, irrespective of background treatment. Studies that combined multiple RCTs (with data of interest not presented in a single RCT) were also included, and those that included non‐RCTs were excluded. Furthermore, reviews, letters, case reports, editorials, commentaries, expert opinions, and meta‐analyses were also excluded. Studies not reporting data of interest with standard errors and/or deviations were excluded. Unpublished data or data published only in abstracts were not used. Secondary information sources, including post hoc studies and subgroup studies, were considered and discussed separately in the event that the main variable or other variables that were not included in the analysis of the first publication of the original trial are analyzed from a sex perspective.

Two independent reviewers (Y.Y. and S.H.) assessed the eligibility of studies for inclusion in the analysis. Any discrepancies were discussed with other team members until a consensus was reached to include only the studies that best fulfilled the criteria.

### 2.3 Outcome Definitions

Differences in weight reduction stratified according to sex were the only outcomes addressed in this systematic review and meta‐analysis. The unit of weight change is defined in two ways: (1) kilograms change from baseline and (2) percentages of body weight change from baseline.

### 2.4 Data Extraction and Quality Assessment

Data were extracted according to trial information (trial name or first author, sample size for each sex, age, body weight, body mass index (BMI), glycated hemoglobin levels, trial duration, GLP‐1 RA administration, indication for treatment, duration of treatment, comparator drugs, and background treatment) and reported outcomes (overall and sex changes in weight from baseline). Two independent reviewers (Y.Y. and S.H.) extracted data from the included studies. Discrepancies were resolved by other team members.

Version 2 of the Cochrane Risk‐of‐Bias Tool for Randomized Trials was used to assess the risk of bias in the outcome (i.e., change in weight) [10]. If a study included multiple RCTs, each RCT was assessed. Trials with a low risk of bias in all domains were considered to have an overall low risk of bias, whereas those with a high risk of bias in ≥ 1 domain(s) were considered to have an overall high risk of bias. Quality assessment was performed by four independent reviewers (Y.Y., L.H., F.P., and H.Z.), and any disagreements were resolved by consensus discussion.

### 2.5 Data Synthesis

Meta‐analyses were performed using an inverse variance random‐effects model to calculate the mean difference (MD) and corresponding 95% confidence interval (CI) for continuous outcomes regardless of *I* ^2^ value. Standard deviation (SD) was calculated from the standard error or 95% CI, according to the Cochrane Handbook for Systematic Reviews of Interventions [11]. The Higgins *I* ^2^ statistic and Cochran's *Q* test were used to assess potential statistical heterogeneity among the trials [12]. *I* ^2^ statistic values > 50% were considered to be indicative of heterogeneity. One trial may have had ≥ 2 different doses of GLP‐1RA treatments. If a study contained > 1 dose of the same GLP‐1 RA, the data for different doses were combined according to the Cochrane Handbook for Systematic Reviews of Interventions [11]. Data for the same type of GLP‐1 RAs were combined to present a pooled sex difference in weight reduction, in accordance with the Cochrane Handbook for Systematic Reviews of Interventions [11]. Meta‐regression analysis was performed to assess the association between weight reduction and sex.

Subgroup analyses were stratified according to individual GLP‐1RA medications, high or low dose of GLP‐1RA (high dose, liraglutide ≥ 1.8 mg once daily; subcutaneous semaglutide ≥ 1.0 mg once weekly; dulaglutide ≥ 1.5 mg once weekly; retatrutide > 1 mg once weekly; vice versa was the low dose), shorter or longer treatment duration (> 26 or ≤ 26 weeks), indication for treatment (type 2 DM or obesity), type of control (placebo or active comparator), background treatment (sulfonylureas or non‐sulfonylureas), and baseline weight (higher weight, heavier than median weight; vice versa was the lower weight). If a study contained > 1 GLP‐1RA dataset, all datasets inside this study were combined into a single dataset in accordance with the Cochrane Handbook for Systematic Reviews of Interventions [11].

Sensitivity analyses were performed when a study reporting substantial weight reduction was conducted to determine the impact of massive effects and validate the robustness of the results.

Publication bias was evaluated by visual assessment of the asymmetry of funnel plots and Egger's asymmetry test [13]. The trim and fill method was applied to the meta‐analysis of included studies to assess the robustness of the results and rectify publication bias.

All statistical analyses were performed using R version 4.2.1 (R Foundation for Statistical Computing, Vienna, Austria) and Stata/SE Release 17.0 (StataCorp. LLC, College Station, TX, USA). Differences with a two‐tailed *p* < 0.05 were considered to be statistically significant.

## 3 Results

### 3.1 Search Results and Study Characteristics

**FIGURE 1:**
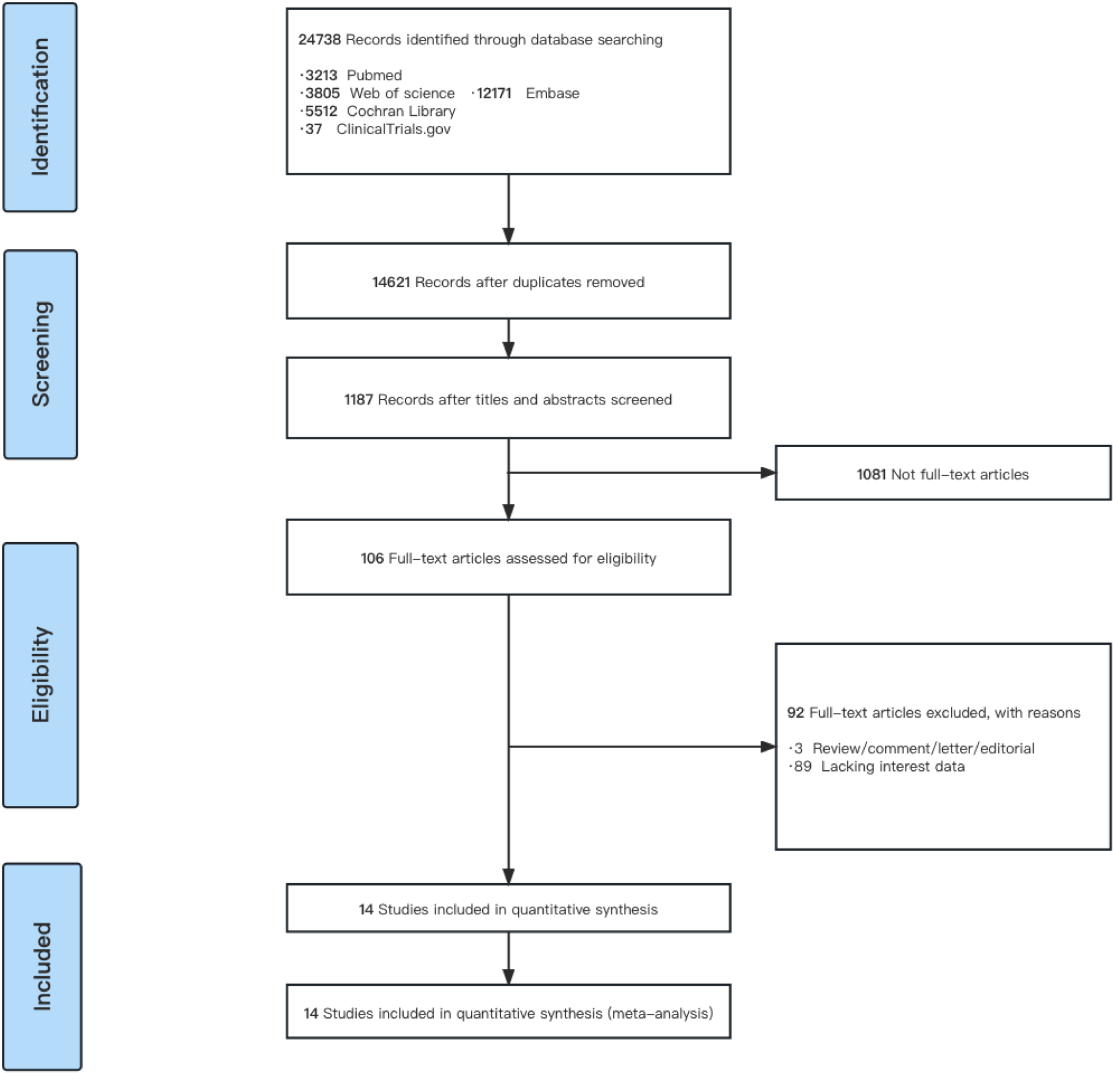
Flowchart of identification and selection of included studies for systematic review and meta‐analysis.

A flow diagram illustrating the literature search process is presented in Figure 1. Overall, 24,307 references were retrieved in the search. After excluding duplicate studies, those that did not fulfill the inclusion criterion and those that fulfilled the exclusion criteria, 14 were eligible for quantitative data synthesis [4, 14, 15, 16, 17, 18, 19, 20, 21, 22, 23, 24, 25, 26]. Five types of GLP‐1 RAs were included in the meta‐analysis (dulaglutide, exenatide, liraglutide, semaglutide, and retatrutide). The eligible trials lasted from 24 to 104 weeks. Of the included studies, 4 reported results of weight reduction with semaglutide, [4, 23, 24, 25] along with 8 studies of dulaglutide, [14, 15, 16, 17, 18, 19, 20, 24] 3 studies of exenatide (one with multiple RCTs), [14, 21, 22] 1 study of retatrutide, [26] and 1 study of liraglutide [19]. The average age of the patients in the included studies was 55.3 years. The average BMI among the included studies was 32.8 kg/m^2^, and the average weight was 91.81 kg. The proportion of females ranged from 37.0% to 66.7%. Characteristics of the included studies are summarized in Table 1. More detailed information regarding the included studies is summarized in Tables S2 and S3.

**TABLE 1:** Study characteristics of included studies.

Trials	Sample size	Treatment duration	Age (year)	Number of males	Number of females	BMI (kg/m^2^)	Weight at baseline (kg)	Interventions	Weight change from baseline (kg)
AWARD‐1 [14]	816	26 weeks	55.66 ± 9.68	477	339	33.34 ± 5.36	96.34 ± 19.99	Exenatide/Dulaglutide	Overall: −0.73 ± 4.82 Male: −0.65 ± 4.52 Female: −0.87 ± 4.38
AWARD‐2 [15]	529	52 weeks	56.50 ± 9.51	274	255	31.50 ± 5.02	85.5 ± 17.99	Dulaglutide	Overall: −1.60 ± 3.91 Male: −1.27 ± 3.13 Female: −1.96 ± 3.09
AWARD‐3 [16]	530	26 weeks	56.00 ± 10.99	229	301	33.50 ± 6.02	92.5 ± 18.99	Dulaglutide	Overall: −1.83 ± 3.93 Male: −1.33 ± 3.61 Female: −2.24 ± 3.77
AWARD‐4 [17]	548	52 weeks	59.10 ± 9.30	286	262	32.55 ± 5.18	91.35 ± 18.09	Dulaglutide	Overall: −0.34 ± 4.50 Male: 0.12 ± 4.08 Female: −0.95 ± 4.27
AWARD‐5 [18]	598	104 weeks	54.00 ± 9.99	275	323	31.00 ± 4.53	86.5 ± 17.5	Dulaglutide	Overall: −2.64 ± 4.38 Male: −2.28 ± 3.12 Female: −3.48 ± 3.20
AWARD‐6 [19]	593	26 weeks	56.65 ± 9.60	284	309	33.55 ± 5.15	94.1 ± 18.6	Liraglutide/Dulaglutide	Overall: −3.26 ± 3.82 Male: −2.64 ± 3.62 Female: −3.73 ± 3.61
AWARD‐8 [20]	236	24 weeks	57.7 ± 10.2	102	134	30.9 ± 5.2	84.5 ± 16.4	Dulaglutide	Overall: −0.91 ± 3.23 Male: −0.24 ± 3.45 Female: −0.75 ± 3.51
DURATION‐1‐6 [21]	1719	24–30 weeks	55 ± 10.29	944	775	31.3 ± 5.7	87.43 ± 20.48	Exenatide	Overall: −2.37 ± 3.34 Male: −2.1 ± 3.14 Female: −2.7 ± 3.55
DURATION‐8 [22]	184	28 weeks	54 ± 10	96	88	32 ± 5.9	89.8 ± 20.2	Exenatide	Overall: −1.55 ± 4.01 Male: −0.56 ± 3.92 Female: −2.63 ± 3.85
Jastreboff 2023 [26]	268	48 weeks	48.2 ± 12.7	139	129	37.3 ± 5.7	107.34 ± 21.5	Retatrutide	Overall: −18.06 ± 10.65 Male: −16.04 ± 9.54 Female: −20.25 ± 10.88
STEP‐1 [25]^a^	228	68 weeks	48 ± 12	76	152	37.6 ± 7.0	105.6 ± 21.8	Semaglutide	Overall: −17.3 ± 9.3 Male: −14.5 ± 8.0 Female: −18.7 ± 9.6
SUSTAIN‐6 [23]	1648	104 weeks	64.65 ± 7.20	1013	635	32.80 ± 6.23	92.35 ± 20.7	Semaglutide	Overall: −4.25 ± 12.34 Male: −3.91 ± 10.62 Female: −4.72 ± 10.76
SUSTAIN‐7 [24]	1199	40 weeks	55.50 ± 10.63	662	537	33.50 ± 6.78	95.23 ± 22.56	Dulaglutide/Semaglutide	Overall: −4.10 ± 5.03 Male: −3.54 ± 8.38 Female: −4.80 ± 8.56
SUSTAIN‐China [4]	578	30 weeks	53.00 ± 11.00	314	264	28.05 ± 5.00	76.85 ± 16.35	Semaglutide	Overall: −3.55 ± 7.14 Male: −2.64 ± 7.07 Female: −3.62 ± 7.10

*Note:* Data were shown as mean ± SD.

Abbreviations: BMI, body mass index; SD, standard deviation.

^a^ The weight change from baseline in STEP‐1 is evaluated using the percentage change in body weight; therefore, the unit is percentage rather than kilograms. This study was not included in the meta‐analysis that used kilograms as the outcome measure.

### 3.2 Risk of Bias Assessment

The risk of bias in the included RCTs is reported in Figure S1. Among these RCTs, there was a high risk in the randomization process in four studies due to study design [19, 24, 27, 28]. There were some concerns about the overall risk of bias [4, 14, 15, 16, 17, 18, 20, 22, 29, 30, 31, 32] although other biases were low in all studies.

### 3.3 Pooled Effect of GLP‐1 RAs and Meta‐Regression Analysis

**FIGURE 2:**
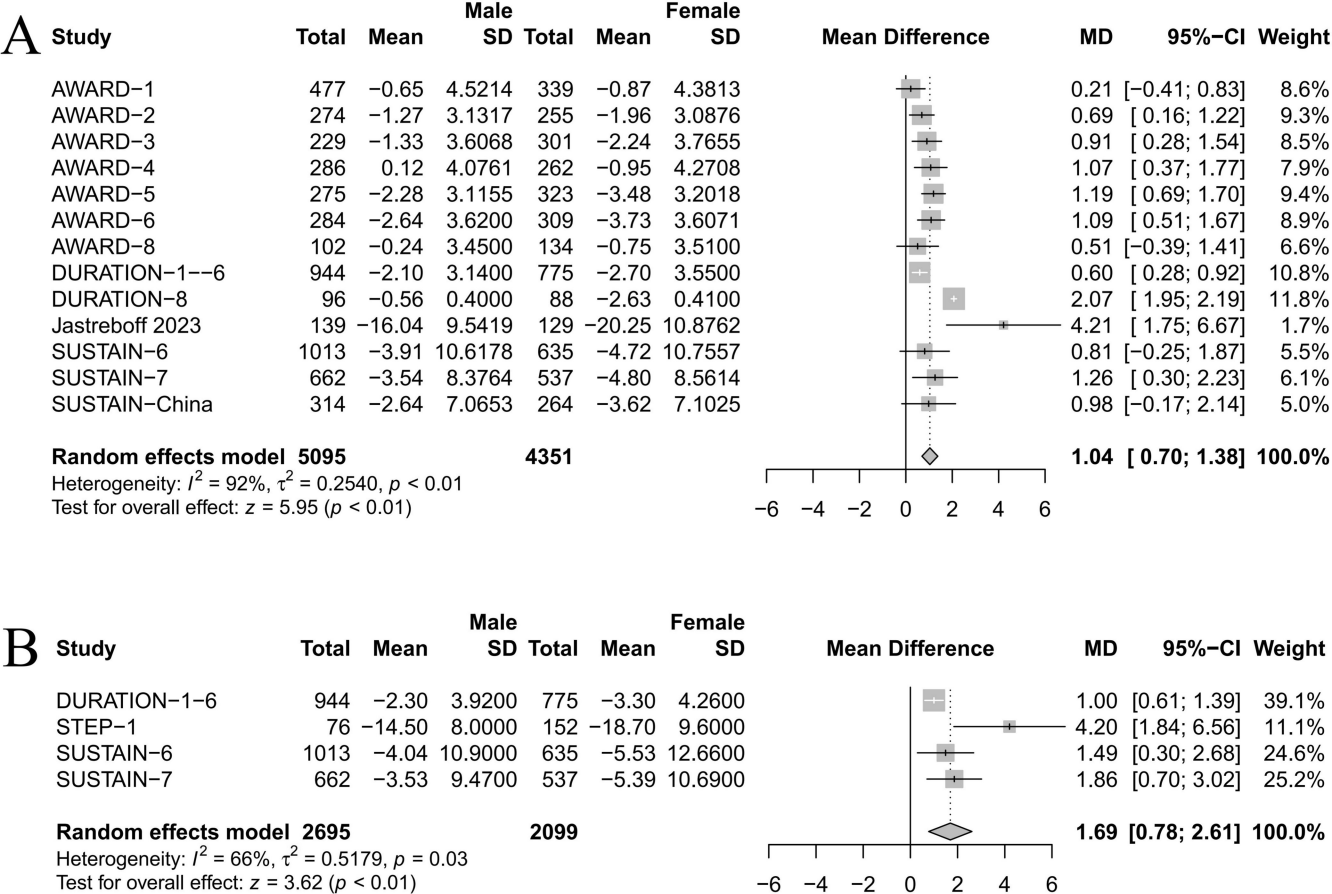
Forest plot for meta‐analysis of included studies. Results were summarized by individual studies. One trial might have over one dose or type of GLP‐1 RA treatments, and the data would be combined to a single dataset. (A) The unit of weight change is the kilogram; (B) the unit of weight change is the percentage. CI, confidential interval.

Meta‐analysis of 13 studies revealed that females lost more weight than males (MD 1.04 kg [95% CI 0.70–1.38], *p* < 0.01) (Figure 2A). Another meta‐analysis of four studies on the percent of weight change also showed that females lost more weight than males (MD 1.69% [95% CI 0.78–2.61], *p* < 0.01) (Figure 2B). According to findings, studies that did not find sex differences in weight reduction efficacy reported an average weight reduction of 2.36 kg. Conversely, studies in which sex differences were evident reported an average weight loss of 3.58 kg.

**FIGURE 3:**
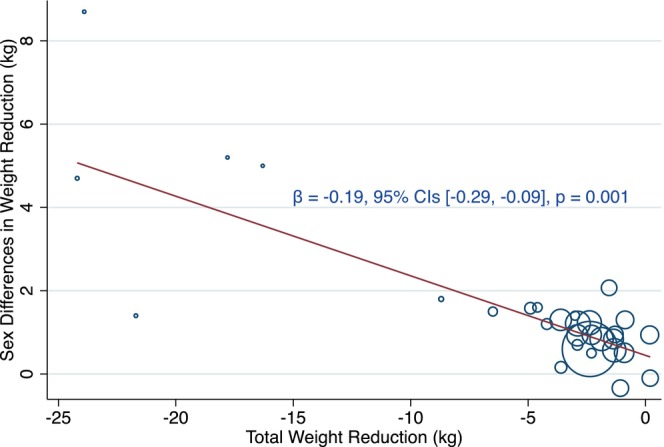
Association between the amount of weight reduction and gender differences in weight reduction of GLP‐1 RAs. Each circle represents individual study data. The size of each circle is indicative of its weight in the meta‐regression analysis and larger circles denote studies with greater influence on the overall result. The vertical axis measures the difference in weight reduction between females and males, with positive values signifying additional weight reduction observed in women compared to men. GLP‐1 RA, glucagon‐like peptide‐1 receptor agonists.

Based on the results of meta‐regression, substantial weight reduction was significantly relevant to greater sex disparities (*β* −0.19 [95% CI −0.29 to −0.09], *p* < 0.01) (Figure 3). Studies with weight reduction > 5% of baseline body weight were further analyzed and revealed that the sex difference was larger (MD 1.22 kg [95% CI 0.63–1.81], *p* < 0.01) (Figure S2).

### 3.4 Subgroup Analysis

Pooled results for individual GLP‐1 RAs indicated that females exhibited more weight reduction than males (MD 0.88 kg [95% CI 0.67–1.09], *p* < 0.01) (Figure S3). With regard to different types of GLP‐1 RAs, dulaglutide, liraglutide, semaglutide, and retatrutide yielded statistically significant differences in weight reduction between males and females (dulaglutide, MD 0.88 kg [95% CI 0.63–1.12], *p* < 0.01; liraglutide, MD 1.30 kg [95% CI 0.48–2.12]; semaglutide, MD 1.04 kg [95% CI 0.45–1.63], *p* < 0.01; retatrutide, MD 4.21 kg [95% CI 1.75–6.67]) (Figure S3). No sex difference was observed in the exenatide subgroup analysis (MD 0.75 kg [95% CI −0.52–2.02], *p* = 0.25) (Figure S3).

Subgroup analysis demonstrated that the sex difference in weight reduction from GLP‐1 RAs was affected by indications for treatment (Figure S4). With indications for weight reduction, the sex difference for GLP‐1 RAs was larger than with indications for type 2 DM (weight reduction: MD 4.21 kg [95% CI 1.75–6.67], *p* < 0.01; type 2 DM: MD 0.99 kg [95% CI 0.65–1.32], *p* < 0.01; *P* for subgroup differences < 0.01) (Figure S5). Background treatment, dose of GLP‐1 RAs, duration of treatment, baseline weight, and type of control had no subgroup differences in the sex difference in weight reduction for GLP‐1 RAs (Figures S5–S9).

Regarding higher or lower doses of individual GLP‐1 RAs, both doses of dulaglutide (low dose: MD 0.76 kg [95% CI 0.38–1.14], *p* < 0.01; high dose: MD 0.97 kg [95% CI 0.65–1.29], *p* < 0.01; *p* for subgroup differences = 0.40), high dose of semaglutide (MD 1.43 kg [95% CI 0.47–2.39], *p* < 0.01; *p* for subgroup differences = 0.30), and high dose of retatrutide (MD 4.86 kg [95% CI 2.50–7.22], *p* < 0.01; *p* for subgroup differences = 0.16) manifested sex differences in weight reduction (Figures S10–S12).

### 3.5 Sensitivity Analysis and Publication Bias

After excluding one study reporting substantial weight reduction, [26] the pooled results remained similar (MD 0.99 kg [95% CI 0.65–1.32], *p* < 0.01) (Figure S13). After excluding retatrutide in the pooled meta‐analysis of GLP‐1 RAs, sex differences in weight reduction were statistically significant (MD 0.85 kg [95% CI 0.65–1.05], *p* < 0.01) (Figure S14).

Publication bias was not observed in the meta‐analysis of included studies according to Egger's test (*p* = 0.09). The funnel plot of the meta‐analysis of the 14 included studies was visually symmetrical (Figure S15).

## 4 Discussion

To the best of our knowledge, this is the first systematic review and meta‐analysis to investigate sex differences in the efficacy of GLP‐1 RAs for weight reduction, with females losing more weight than males. Sex‐based differences in weight reduction became more pronounced as the degree of weight reduction increased. Moreover, the sex difference was greater in trials for weight reduction and longer treatment duration. Sensitivity analysis indicated that the results were robust.

Our results revealed a discernible sex difference in the efficacy of GLP‐1 RAs for weight reduction, despite the fact that labels for GLP‐1 RAs describe no observable sex differences [5, 6, 7, 8]. Labeling for semaglutide states, “The efficacy of Ozempic was not impacted by age, gender, race, ethnicity, BMI at baseline, and body weight (kg) at baseline.” [5] Although some studies have identified sex differences in the efficacy of GLP‐1 RAs for weight reduction, [2, 33, 34, 35, 36] others have reported comparable weight reduction effects in both sexes treated with GLP‐1 RAs [3, 4]. Inconsistent findings among previous studies investigating the efficacy of GLP‐1 RAs could be attributed to variations in study settings, including diverse treatment indications and durations, insufficient sample sizes, as well as potential confounding factors. Notably, our research revealed that sex differences became more pronounced as the degree of weight reduction increased, which could potentially account for why previous studies failed to detect any sex differences due to insufficient weight reduction effects. According to our findings, studies that did not report sex differences in weight reduction efficacy reported an average weight reduction of 2.36 kg, while those in which sex differences were evident yielded an average weight loss of 3.58 kg. Although the difference in the efficacy of GLP‐1 RAs for weight reduction between females and males was 1.04 kg, this difference increased to 1.22 kg in those with a weight reduction > 5% of baseline body weight. The extent of weight reduction after retatrutide (4/8 mg) treatment reached 23.9 kg, and the difference in weight loss between females and males was 8.7 kg [26]. Even a small weight reduction can reduce the risk of developing DM [37]. In the context of impaired glucose tolerance, every 1 kg of body weight reduction corresponds to a notable 16% decline in the likelihood of developing DM, as well as favorable changes in lipid profiles and blood pressure [38, 39]. Furthermore, women typically have a lower baseline body weight and, thus, higher absolute weight reduction translates into a proportionally larger percentage of overall body weight reduction [40].

Previous studies have confirmed that there is no sex difference in glycemic control or the incidence of major adverse cardiac events (MACE) associated with GLP‐1 RAs [33, 34, 41, 42, 43]. Thus, there was no sex difference between GLP‐1 RAs in glycemic control and MACE, while a sex difference was found in weight reduction, possibly due to a separate mechanism(s) [44]. The inconsistency in observed sex differences between weight reduction and MACE outcomes may stem from the possibility that the achieved weight reductions were not sufficiently substantial to yield a discernible influence on CVD endpoints or that a longer duration of sustained weight reduction is necessary for its effects to manifest in MACE [45, 46]. Moreover, considering the increased sex differences that accompany greater degrees of weight reduction, conducting sex‐specific analyses of glycemic control, blood pressure, steatohepatitis, MACE, and additional pertinent health metrics within GLP‐1 RAs research featuring substantial weight reduction outcomes would be warranted.

Several specific subgroups have been found to yield more substantial weight reduction, such as obesity‐specific therapy indications. This observation suggests that baseline patient characteristics could affect the prediction of potential sex differences in weight reduction outcomes. The degree of weight reduction in trials of GLP‐1 RAs for obesity treatment was higher than that in DM treatment. Subgroup analyses stratified according to GLP‐1 RA dose, baseline body weight, duration of treatment, background treatment, and type of control revealed that sex differences were also observed in spite of no subgroup differences. In particular, the absolute weight reduction in high and low doses of GLP‐1 RAs was 1.10 and 0.76 kg (Figure S7), along with the weight reduction of a high or low dose of semaglutide 1.43 and 0.71 kg (Figure S11). Despite no observed sex‐based subgroup differences in weight reduction between high and low doses, our study found different values of weight reduction at high and low doses, which were 1.10 and 0.76 kg for GLP‐1 RAs (Figure S7) and 1.43 and 0.71 kg for the semaglutide group (Figure S11), with only marginal differences of 0.34 and 0.72 kg, respectively. As mentioned, the difference in weight reduction between males and females was positively correlated with overall weight reduction. Hence, the current standard approaches to studying low‐ and high‐dose regimens may fail to discern sex‐based differences in weight reduction, especially with relatively insufficient efficacy for weight reduction. It is noteworthy that a tendency for sex differences was observed among different doses, although this was not statistically significant, possibly due to the limited number of studies, relatively small sample sizes, and most importantly, the insufficient degree of weight reduction.

Sex may play a vital role in the weight reduction efficacy of GLP‐1 RAs via multiple mechanisms. First, sex differences in pharmacokinetics contributing to higher plasma levels of GLP‐1 RAs in females may be responsible for greater weight reduction in females compared with males [41, 47, 48]. Increased exposure to GLP‐1 RAs was observed in females owing to the lower weight of females compared with males [40, 49]. Decreased clearance of GLP‐1 RAs was observed in females compared with weight‐matched males [47]. Second, sex hormones may play a role in this difference [50, 51]. GLP‐1 RAs and estrogen could act synergistically to activate the supramammillary nucleus, which controls food intake, inducing a greater modification of the food‐reward behavior through pro‐opiomelanocortin neurons, thus a greater reduction in weight [52, 53]. Moreover, the combination of GLP‐1 RAs and estrogen may ameliorate leptin resistance, which contributes to greater weight reduction [53]. Third, the more significant improvement in obesity‐related biomarkers and adipocytokine levels, such as C‐reactive protein and tumor necrosis factor‐alpha, among females compared with males undergoing GLP‐1 RA therapy potentially explains their enhanced effectiveness in achieving weight reduction because a decrease in these markers may be linked to the observed weight loss, which is known to contribute to sex‐based differences in weight reduction outcomes [2]. Fourth, GLP‐1 RAs may cause more gastrointestinal adverse reactions in females than in males, which may also contribute to a lower energy intake [49]. Finally, more stringent adherence to GLP‐1 RAs among females may also, in part, account for this difference [2].

The present study had several strengths. To the best of our knowledge, it was the first to systematically confirm sex differences in the efficacy of GLP‐1 RAs for weight reduction. Second, in addition to sex differences, we further revealed that the difference increased with greater weight reduction. Third, we subsequently performed subgroup analyses to identify whether baseline characteristics play a role in this sex difference, which was helpful in anticipating sex differences in the use of GLP‐1 RAs in clinical practice.

However, our study also had several limitations. First, the number of studies and types of GLP‐1 RAs included in our analysis were limited due to the lack of sex‐specific data in numerous studies, such as other dual or triple hormone agonists. Although we searched all GLP‐1‐related medications, including single, dual, and triple hormone agonists, along with oral GLP‐1 RAs like orforglipron and danuglipron, we only found data on a triple hormone receptor agonist, retatrutide. Our results remained consistent when the analysis was exclusively confined to GLP‐1 RAs. Second, we incorporated post hoc analyses in our meta‐analysis, which may have led to an increased risk of bias. Moreover, some studies only provided pooled data from several studies rather than individual data from each, although the data were not reused. Third, the available data represent absolute weight reduction instead of percentage of weight reduction, making it difficult to evaluate relative sex differences in the efficacy of GLP‐1 RAs for weight reduction from initial body weight. Fourth, confounders, including adherence, gastrointestinal adverse reactions, lifestyle and diet changes, as well as baseline BMI or weight between females and males, were not available to be evaluated in this study. Finally, the risk of publication bias was inevitable due to the lack of sex‐specific data in lots of studies; although publication bias was not detected; therefore, conclusions must be drawn with caution. Further investigation of sex differences in GLP‐1 RAs is warranted.

## 5 Conclusions

Females and males reacted differently to treatment with GLP‐1 RAs for weight reduction, with females losing more weight than males. The sex difference in weight reduction became more pronounced as the degree of weight reduction increased. Indications for obesity could magnify this sex difference. Future research could explore the possible mechanism(s) underlying this difference, which would be tremendously beneficial for a more precise prescription of GLP‐1 RAs. We recommend that future research investigating GLP‐1 RAs should meticulously report sex‐specific variations in weight reduction, as well as other outcomes, with the aim of more effectively evaluating the impact of sex differences in weight reduction on diverse health outcomes.

## Author Contributions

H.Z. and Y.L. are joint corresponding authors and contributed equally to this work. H.Z., Y.Y., S.H., and Y.L. conceived and designed the study. Y.Y. and S.H. did the relevant literature searches. Y.Y. and S.H. screened the articles for inclusion. Y.Y. and S.H. extracted the data for analysis. Y.Y., L.H., and S.H. were involved in the quality assessment of the eligible studies. Y.Y. and L.H. did the meta‐analysis and produced forest plots and summary results under the supervision of H.Z. and Y.L. Y.Y., L.H., and H.Z. drafted the manuscript. F.P., Y.L., X.W., N.Y., Z.L., W.L., L.X., H.Z., and Y.L. critically revised the manuscript for important intellectual content. All authors approved the final manuscript. H.Z. and Y.L. are the guarantors. The corresponding authors (H.Z. and Y.L.) attest that all listed authors meet the authorship criteria and that no others, who meet the criteria have been omitted.

## Ethics Statement

The authors have nothing to report.

## Conflicts of Interest

The authors declare no conflicts of interest.

## Supporting information


**Data S1.** Supporting information.

## Data Availability

The study‐specific summary data included in the meta‐analysis can be obtained from the corresponding authors at huabingzhangchn@163.com or liyuxiu@medmail.com.cn. The lead authors (the manuscript's guarantors) affirm that the manuscript is an honest, accurate, and transparent account of the study being reported; that no important aspects of the study have been omitted; and that any discrepancies from the study as planned (and, if relevant, registered) have been explained.

## References

[1] X. Sheng , X. Ye , X. Shi , et al., “A Combination of Plasma Exchange and Steroids in the Treatment of a‐Lipoic Acid‐Induced Insulin Autoimmune Syndrome,” Endokrynologia Polska 72, no. 1 (2021): 81–82. 33295634 10.5603/EP.a2020.0085

[2] H. Quan , H. Zhang , W. Wei , and T. Fang , “Gender‐Related Different Effects of a Combined Therapy of Exenatide and Metformin on Overweight or Obesity Patients With Type 2 Diabetes Mellitus,” Journal of Diabetes and its Complications 30, no. 4 (2016): 686–692. 26873871 10.1016/j.jdiacomp.2016.01.013

[3] R. E. Pratley , V. R. Aroda , A.‐M. Catarig , et al., “Impact of Patient Characteristics on Efficacy and Safety of Once‐Weekly Semaglutide Versus Dulaglutide: SUSTAIN 7 Post Hoc Analyses,” BMJ Open 10, no. 11 (2020): e037883. 10.1136/bmjopen-2020-037883 PMC7670946 33199417

[4] L. Ji , X. Dong , Y. Li , et al., “Efficacy and Safety of Once‐Weekly Semaglutide Versus Once‐Daily Sitagliptin as Add‐On to Metformin in Patients With Type 2 Diabetes in SUSTAIN China: A 30‐Week, Double‐Blind, Phase 3a, Randomized Trial,” Diabetes, Obesity and Metabolism 23, no. 2 (2021): 404–414. 33074557 10.1111/dom.14232 PMC7839591

[5] U.S. Food and Drug Administration , “Full Prescribing Information for Ozempic,” (2017).

[6] U.S. Food and Drug Administration , “Full Prescribing Information for Trulicity,” (2014).

[7] U.S. Food and Drug Administration , “Full Prescribing Information for Victoza,” (2010).

[8] U.S. Food and Drug Administration , “Full Prescribing Information for BYETTA,” (2005).

[9] M. J. Page , J. E. McKenzie , P. M. Bossuyt , et al., “The PRISMA 2020 Statement: An Updated Guideline for Reporting Systematic Reviews,” BMJ 372 (2021): n71. 33782057 10.1136/bmj.n71 PMC8005924

[10] J. A. C. Sterne , J. Savović , M. J. Page , et al., “RoB 2: A Revised Tool for Assessing Risk of Bias in Randomised Trials,” British Medical Journal (Clinical Research Edition) 366 (2019): l4898. 10.1136/bmj.l4898 31462531

[11] J. P. T. Higgins , J. Thomas , J. Chandler , et al., eds., Cochrane Handbook for Systematic Reviews of Interventions Version 6.4 (Cochrane, 2023).

[12] J. P. T. Higgins and S. G. Thompson , “Quantifying Heterogeneity in a Meta‐Analysis,” Statistics in Medicine 21, no. 11 (2002): 1539–1558. 12111919 10.1002/sim.1186

[13] M. Egger , G. Davey Smith , M. Schneider , and C. Minder , “Bias in Meta‐Analysis Detected by a Simple, Graphical Test,” British Medical Journal (Clinical Research Edition) 315, no. 7109 (1997): 629–634. 10.1136/bmj.315.7109.629 PMC2127453 9310563

[14] C. Wysham , T. Blevins , R. Arakaki , et al., “Efficacy and Safety of Dulaglutide Added Onto Pioglitazone and Metformin Versus Exenatide in Type 2 Diabetes in a Randomized Controlled Trial (AWARD‐1),” Diabetes Care 37, no. 8 (2014): 2159–2167. 24879836 10.2337/dc13-2760

[15] F. Giorgino , M. Benroubi , J. H. Sun , A. G. Zimmermann , and V. Pechtner , “Efficacy and Safety of Once‐Weekly Dulaglutide Versus Insulin Glargine in Patients With Type 2 Diabetes on Metformin and Glimepiride (AWARD‐2),” Diabetes Care 38, no. 12 (2015): 2241–2249. 26089386 10.2337/dc14-1625

[16] G. Umpierrez , S. Tofe Povedano , F. Perez Manghi , L. Shurzinske , and V. Pechtner , “Efficacy and Safety of Dulaglutide Monotherapy Versus Metformin in Type 2 Diabetes in a Randomized Controlled Trial (AWARD‐3),” Diabetes Care 37, no. 8 (2014): 2168–2176. 24842985 10.2337/dc13-2759

[17] L. Blonde , J. Jendle , J. Gross , et al., “Once‐Weekly Dulaglutide Versus Bedtime Insulin Glargine, Both in Combination With Prandial Insulin Lispro, in Patients With Type 2 Diabetes (AWARD‐4): A Randomised, Open‐Label, Phase 3, Non‐Inferiority Study,” Lancet 385, no. 9982 (2015): 2057–2066. 26009229 10.1016/S0140-6736(15)60936-9

[18] R. S. Weinstock , B. Guerci , G. Umpierrez , M. A. Nauck , Z. Skrivanek , and Z. Milicevic , “Safety and Efficacy of Once‐Weekly Dulaglutide Versus Sitagliptin After 2 Years in Metformin‐Treated Patients With Type 2 Diabetes (AWARD‐5): A Randomized, Phase III Study,” Diabetes, Obesity & Metabolism 17, no. 9 (2015): 849–858. 25912221 10.1111/dom.12479 PMC5008205

[19] K. M. Dungan , S. T. Povedano , T. Forst , et al., “Once‐Weekly Dulaglutide Versus Once‐Daily Liraglutide in Metformin‐Treated Patients With Type 2 Diabetes (AWARD‐6): A Randomised, Open‐Label, Phase 3, Non‐Inferiority Trial,” Lancet 384, no. 9951 (2014): 1349–1357. 25018121 10.1016/S0140-6736(14)60976-4

[20] K. M. Dungan , R. Weitgasser , F. Perez Manghi , et al., “A 24‐Week Study to Evaluate the Efficacy and Safety of Once‐Weekly Dulaglutide Added on to Glimepiride in Type 2 Diabetes (AWARD‐8),” Diabetes, Obesity & Metabolism 18, no. 5 (2016): 475–482. 26799540 10.1111/dom.12634 PMC5067625

[21] R. Pencek , A. Blickensderfer , Y. Li , S. C. Brunell , and S. Chen , “Exenatide Once Weekly for the Treatment of Type 2 Diabetes: Effectiveness and Tolerability in Patient Subpopulations,” International Journal of Clinical Practice 66, no. 11 (2012): 1021–1032. 22925173 10.1111/j.1742-1241.2012.03006.x PMC3506736

[22] J. P. Frias , E. Hardy , A. Ahmed , et al., “Effects of Exenatide Once Weekly Plus Dapagliflozin, Exenatide Once Weekly Alone, or Dapagliflozin Alone Added to Metformin Monotherapy in Subgroups of Patients With Type 2 Diabetes in the DURATION‐8 Randomized Controlled Trial,” Diabetes, Obesity & Metabolism 20, no. 6 (2018): 1520–1525. 29573139 10.1111/dom.13296 PMC5969323

[23] S. P. Marso , S. C. Bain , A. Consoli , et al., “Semaglutide and Cardiovascular Outcomes in Patients With Type 2 Diabetes,” New England Journal of Medicine 375, no. 19 (2016): 1834–1844. 27633186 10.1056/NEJMoa1607141

[24] R. E. Pratley , V. R. Aroda , I. Lingvay , et al., “Semaglutide Versus Dulaglutide Once Weekly in Patients With Type 2 Diabetes (SUSTAIN 7): A Randomised, Open‐Label, Phase 3b Trial,” Lancet Diabetes and Endocrinology 6, no. 4 (2018): 275–286. 29397376 10.1016/S2213-8587(18)30024-X

[25] J. P. H. Wilding , R. L. Batterham , M. Davies , et al., “Weight Regain and Cardiometabolic Effects After Withdrawal of Semaglutide: The STEP 1 Trial Extension,” Diabetes, Obesity & Metabolism 24, no. 8 (2022): 1553–1564. 35441470 10.1111/dom.14725 PMC9542252

[26] A. M. Jastreboff , L. M. Kaplan , J. P. Frias , et al., “Retatrutide Phase 2 Obesity Trial I. Triple‐Hormone‐Receptor Agonist Retatrutide for Obesity ‐ A Phase 2 Trial,” New England Journal of Medicine 389, no. 6 (2023): 514–526. 37366315 10.1056/NEJMoa2301972

[27] T. Blevins , J. Pullman , J. Malloy , et al., “DURATION‐5: Exenatide Once Weekly Resulted in Greater Improvements in Glycemic Control Compared With Exenatide Twice Daily in Patients With Type 2 Diabetes,” Journal of Clinical Endocrinology and Metabolism 96, no. 5 (2011): 1301–1310. 21307137 10.1210/jc.2010-2081

[28] D. J. Drucker , J. B. Buse , K. Taylor , et al., “Exenatide Once Weekly Versus Twice Daily for the Treatment of Type 2 Diabetes: A Randomised, Open‐Label, Non‐Inferiority Study,” Lancet 372, no. 9645 (2008): 1240–1250. 18782641 10.1016/S0140-6736(08)61206-4

[29] R. M. Bergenstal , C. Wysham , L. Macconell , et al., “Efficacy and Safety of Exenatide Once Weekly Versus Sitagliptin or Pioglitazone as an Adjunct to Metformin for Treatment of Type 2 Diabetes (DURATION‐2): A Randomised Trial,” Lancet 376, no. 9739 (2010): 431–439. 20580422 10.1016/S0140-6736(10)60590-9

[30] M. Diamant , L. Van Gaal , S. Stranks , et al., “Once Weekly Exenatide Compared With Insulin Glargine Titrated to Target in Patients With Type 2 Diabetes (DURATION‐3): An Open‐Label Randomised Trial,” Lancet (London, England) 375, no. 9733 (2010): 2234–2243. 20609969 10.1016/S0140-6736(10)60406-0

[31] D. Russell‐Jones , R. M. Cuddihy , M. Hanefeld , et al., “Efficacy and Safety of Exenatide Once Weekly Versus Metformin, Pioglitazone, and Sitagliptin Used as Monotherapy in Drug‐Naive Patients With Type 2 Diabetes (DURATION‐4): A 26‐Week Double‐Blind Study,” Diabetes Care 35, no. 2 (2012): 252–258. 22210563 10.2337/dc11-1107 PMC3263915

[32] J. B. Buse , M. Nauck , T. Forst , et al., “Exenatide Once Weekly Versus Liraglutide Once Daily in Patients With Type 2 Diabetes (DURATION‐6): A Randomised, Open‐Label Study,” Lancet 381, no. 9861 (2013): 117–124. 23141817 10.1016/S0140-6736(12)61267-7

[33] B. Gallwitz , S. Dagogo‐Jack , V. Thieu , et al., “Effect of Once‐Weekly Dulaglutide on Glycated Haemoglobin (HbA1c) and Fasting Blood Glucose in Patient Subpopulations by Gender, Duration of Diabetes and Baseline HbA1c,” Diabetes, Obesity & Metabolism 20, no. 2 (2018): 409–418. 28817231 10.1111/dom.13086 PMC6084353

[34] Y. Onishi , T. Oura , A. Matsui , J. Matsuura , and N. Iwamoto , “Analysis of Efficacy and Safety of Dulaglutide 0.75 Mg Stratified by Sex in Patients With Type 2 Diabetes in 2 Randomized, Controlled Phase 3 Studies in Japan,” Endocrine Journal 64, no. 5 (2017): 553–560. 28367916 10.1507/endocrj.EJ16-0552

[35] A. Kautzky‐Willer and J. Harreiter , “Sex and Gender Differences in Therapy of Type 2 Diabetes,” Diabetes Research and Clinical Practice 131 (2017): 230–241. 28779681 10.1016/j.diabres.2017.07.012

[36] R. Anichini , S. Cosimi , A. Di Carlo , et al., “Gender Difference in Response Predictors After 1‐Year Exenatide Therapy Twice Daily in Type 2 Diabetic Patients: A Real World Experience,” Diabetes, Metabolic Syndrome and Obesity 6 (2013): 123–129. 23630427 10.2147/DMSO.S42729 PMC3626369

[37] K. I. Galaviz , M. B. Weber , A. Straus , J. S. Haw , K. M. V. Narayan , and M. K. Ali , “Global Diabetes Prevention Interventions: A Systematic Review and Network Meta‐Analysis of the Real‐World Impact on Incidence, Weight, and Glucose,” Diabetes Care 41, no. 7 (2018): 1526–1534. 29934481 10.2337/dc17-2222 PMC6463613

[38] R. F. Hamman , R. R. Wing , S. L. Edelstein , et al., “Effect of Weight Loss With Lifestyle Intervention on Risk of Diabetes,” Diabetes Care 29, no. 9 (2006): 2102–2107. 16936160 10.2337/dc06-0560 PMC1762038

[39] J. W. Anderson and E. C. Konz , “Obesity and Disease Management: Effects of Weight Loss on Comorbid Conditions,” Obesity Research 9, no. Suppl 4 (2001): 326S–334S. 11707561 10.1038/oby.2001.138

[40] M. Jensterle , M. Rizzo , and A. Janež , “Semaglutide in Obesity: Unmet Needs in Men,” Diabetes Therapy 14, no. 3 (2023): 461–465. 36609945 10.1007/s13300-022-01360-7 PMC9981825

[41] A. K. Singh and R. Singh , “Gender Difference in Cardiovascular Outcomes With SGLT‐2 Inhibitors and GLP‐1 Receptor Agonist in Type 2 Diabetes: A Systematic Review and Meta‐Analysis of Cardio‐Vascular Outcome Trials,” Diabetes and Metabolic Syndrome: Clinical Research and Reviews 14, no. 3 (2020): 181–187. 10.1016/j.dsx.2020.02.012 32142999

[42] A. Diallo , M. Carlos‐Bolumbu , and F. Galtier , “Age, Sex, Race, BMI, and Duration of Diabetes Differences in Cardiovascular Outcomes With Glucose Lowering Drugs in Type 2 Diabetes: A Systematic Review and Meta‐Analysis,” EClinicalMedicine 54 (2022): 101697. 36263397 10.1016/j.eclinm.2022.101697 PMC9574412

[43] E. D'Andrea , A. S. Kesselheim , J. M. Franklin , E. H. Jung , S. P. Hey , and E. Patorno , “Heterogeneity of Antidiabetic Treatment Effect on the Risk of Major Adverse Cardiovascular Events in Type 2 Diabetes: A Systematic Review and Meta‐Analysis,” Cardiovascular Diabetology 19, no. 1 (2020): 154. 32993654 10.1186/s12933-020-01133-1 PMC7525990

[44] M. A. Nauck , D. R. Quast , J. Wefers , and J. J. Meier , “GLP‐1 Receptor Agonists in the Treatment of Type 2 Diabetes – State‐of‐the‐Art,” Molecular Metabolism 46 (2021): 101102. 33068776 10.1016/j.molmet.2020.101102 PMC8085572

[45] E. Gregg , J. Jakicic , G. Blackburn , et al., “Association of the Magnitude of Weight Loss and Changes in Physical Fitness With Long‐Term Cardiovascular Disease Outcomes in Overweight or Obese People With Type 2 Diabetes: A Post‐Hoc Analysis of the Look AHEAD Randomised Clinical Trial,” Lancet Diabetes and Endocrinology 4, no. 11 (2016): 913–921. 27595918 10.1016/S2213-8587(16)30162-0 PMC5094846

[46] R. R. Wing , “Long‐Term Effects of a Lifestyle Intervention on Weight and Cardiovascular Risk Factors in Individuals With Type 2 Diabetes Mellitus: Four‐Year Results of the Look AHEAD Trial,” Archives of Internal Medicine 170, no. 17 (2010): 1566–1575. 20876408 10.1001/archinternmed.2010.334 PMC3084497

[47] F. Mauvais‐Jarvis , H. K. Berthold , I. Campesi , et al., “Sex‐ and Gender‐Based Pharmacological Response to Drugs,” Pharmacological Reviews 73, no. 2 (2021): 730–762. 33653873 10.1124/pharmrev.120.000206 PMC7938661

[48] J. P. H. Wilding , R. V. Overgaard , L. V. Jacobsen , C. B. Jensen , and C. W. le Roux , “Exposure‐Response Analyses of Liraglutide 3.0 Mg for Weight Management,” Diabetes, Obesity & Metabolism 18, no. 5 (2016): 491–499. 26833744 10.1111/dom.12639 PMC5069568

[49] E. Rentzeperi , S. Pegiou , T. Koufakis , M. Grammatiki , and K. Kotsa , “Sex Differences in Response to Treatment With Glucagon‐Like Peptide 1 Receptor Agonists: Opportunities for a Tailored Approach to Diabetes and Obesity Care,” Journal of Personalized Medicine 12, no. 3 (2022): 454. 35330453 10.3390/jpm12030454 PMC8950819

[50] L. Asarian and N. Geary , “Sex Differences in the Physiology of Eating,” American Journal of Physiology. Regulatory, Integrative and Comparative Physiology 305, no. 11 (2013): R1215–R1267. 23904103 10.1152/ajpregu.00446.2012 PMC3882560

[51] K. A. Kerstetter , M. A. Ballis , S. Duffin‐Lutgen , A. E. Carr , A. M. Behrens , and T. E. Kippin , “Sex Differences in Selecting Between Food and Cocaine Reinforcement Are Mediated by Estrogen,” Neuropsychopharmacology 37, no. 12 (2012): 2605–2614. 22871910 10.1038/npp.2012.99 PMC3473343

[52] H. Vogel , S. Wolf , C. Rabasa , et al., “GLP‐1 and Estrogen Conjugate Acts in the Supramammillary Nucleus to Reduce Food‐Reward and Body Weight,” Neuropharmacology 110, no. Pt A (2016): 396–406. 27496691 10.1016/j.neuropharm.2016.07.039

[53] B. Finan , B. Yang , N. Ottaway , et al., “Targeted Estrogen Delivery Reverses the Metabolic Syndrome,” Nature Medicine 18, no. 12 (2012): 1847–1856. 10.1038/nm.3009 PMC3757949 23142820

