# Supplementary material for: Sex Differences in the Efficacy of Glucagon‐Like Peptide‐1 Receptor Agonists for Weight Reduction: A Systematic Review and Meta‐Analysis
Source: J Diabetes. 2025 Mar 5;17(3):e70063. doi: 10.1111/1753-0407.70063 (PMC11880690; doi:10.1111/1753-0407.70063)

**Supplementary Material**

**Sex differences in the efficacy of glucagon-like peptide-1 receptor agonists for weight reduction: A systematic review and meta-analysis**

Yucheng Yang^,^, MM; Liyun He^,^, MD; Shumeng Han^,^, MD; Na Yang^,^, MD; Yiwen Liu^,^, MD; Xuechen Wang^,^, MD; Ziyi Li^,^, PhD; Fan Ping^,^, MD; Lingling Xu^,^, MD; Wei Li^,^, MD; Huabing Zhang*^,^, MD; Yuxiu Li*^,^, MD

**Contents**

**Table S1** Data sources and search strategies in the meta-analysis

**Table S2** Baseline characteristics of included studies

**Table S3** Detailed characteristics of included studies

Figure S1 Risk of Bias version 2 (RoB v2) for assessment of clinical trial studies

**Figure S2** Forest plot for meta-analysis of studies with weight reductions more than 5% of baseline body weight

**Figure S3** Forest plot for meta-analysis of individual GLP-1 RAs

Figure S4 Effects of indication for treatment on the gender difference in weight reduction of GLP-1 RAs

Figure S5 Effects of background treatment on the gender difference in weight reduction of GLP-1 RAs

Figure S6 Effects of different doses on the gender difference in weight reduction of GLP-1 RAs

Figure S7 Effects of duration of treatment on the gender difference in weight reduction of GLP-1 RAs

Figure S8 Effects of baseline body weight on the gender difference in weight reduction of GLP-1 RAs

Figure S9 Effects of type of control on the gender difference in weight reduction of GLP-1 RAs

Figure S10 Effects of different doses on the gender difference in weight reduction of dulaglutide

Figure S11 Effects of different doses on the gender difference in weight reduction of semaglutide

Figure S12 Effects of different doses on the gender difference in weight reduction of retatrutide

**Figure S13** Sensitivity analysis for pooled results of included studies excluding two with substantial weight reduction

Figure S14 Sensitivity analysis for pooled results of GLP-1 RAs excluding retatrutide

**Figure S15** Funnel plot for meta-analysis results of included studies

**Table S1** Data sources and search strategies in the meta-analysis

| **Databases** | **Search number** | **Query** | **Results** |
| --- | --- | --- | --- |
| **PubMed**  **3213** | #1 | Glucagon-Like Peptide-1Receptor' OR 'glucagon like peptide 1 receptor agonist' OR 'glp 1 agonist' OR 'glp 1 receptor agonist' OR 'glucagon like peptide 1 agonist' OR 'glucagon like peptide 1 receptor stimulating agent' OR 'long acting glp 1 agonist' OR 'long acting glp 1 receptor agonist' OR 'long acting glucagon like peptide 1 agonist' OR 'long acting glucagon like peptide 1 receptor agonist' OR 'dulaglutide' OR 'liraglutide' OR 'exenatide' OR 'albiglutide' OR 'semaglutide' OR 'lixisenatide' OR 'tirzepatide' OR ‘cotadutide’ OR ‘efpeglenatide’ OR ‘orforglipron’ OR ‘taspoglutide’ | 14,093 |
|  | #2 | "randomi?ed trial" OR "clinical trial" OR "randomi?ed" OR "random allocation" OR random* OR "controlled trial" OR "randomized controlled trial" | 2,151,772 |
|  | #3 | retraction of publication or retracted publication | 38,779 |
|  | #4 | subgroup OR subpopulation or "post hoc" or post-hoc or substudy or subanalysis or subset or substrates or subtype or integrated | 3,131,517 |
|  | #5 | #2 OR #3 OR #4 | 5,035,007 |
|  | #6 | #1 AND #5 | 3,213 |
| **Embase**  **12171** | #1 | Glucagon-Like Peptide-1Receptor' OR 'glucagon like peptide 1 receptor agonist' OR 'glp 1 agonist'  OR 'glp 1 receptor agonist'  OR 'glucagon like peptide 1 agonist'  OR 'glucagon like peptide 1 receptor stimulating agent'  OR 'long acting glp 1 agonist'  OR 'long acting glp 1 receptor agonist'  OR 'long acting glucagon like peptide 1 agonist'  OR 'long acting glucagon like peptide 1 receptor agonist'  OR 'dulaglutide' OR 'liraglutide' OR 'exenatide' OR 'albiglutide' OR 'semaglutide' OR 'lixisenatide' OR 'tirzepatide' OR ' cotadutide' OR 'efpeglenatide' OR 'orforglipron' OR ' taspoglutide' | 17519 |
|  | #2 | "randomi?ed trial" OR "clinical trial" OR "randomi?ed" OR "random allocation" OR random* OR "controlled trial" OR "randomized controlled trial" | 2545220 |
|  | #3 | retraction of publication or retracted publication | 3550 |
|  | #4 | subgroup OR subpopulation or "post hoc" or post-hoc or substudy or subanalysis or subset or substrates or subtype or integrated | 1391790 |
|  | #5 | #2 OR #3 OR #4 | 3806563 |
|  | #6 | #1 AND #5 | 12171 |
| **Web of science**  **3806** | #1 | TS= ('Glucagon-Like Peptide-1Receptor' OR 'glucagon like peptide 1 receptor agonist' OR 'glp 1 agonist'  OR 'glp 1 receptor agonist'  OR 'glucagon like peptide 1 agonist'  OR 'glucagon like peptide 1 receptor stimulating agent'  OR 'long acting glp 1 agonist'  OR 'long acting glp 1 receptor agonist'  OR 'long acting glucagon like peptide 1 agonist'  OR 'long acting glucagon like peptide 1 receptor agonist'  OR 'dulaglutide' OR 'liraglutide' OR 'exenatide' OR 'albiglutide' OR 'semaglutide' OR 'lixisenatide' OR 'tirzepatide' OR ‘cotadutide’ OR ‘efpeglenatide’ OR ‘orforglipron’ OR ‘taspoglutide’) | 23334 |
|  | #2 | TS=("randomi?ed trial" OR "clinical trial" OR "randomi?ed" OR "random allocation" OR random* OR "controlled trial" OR "randomized controlled trial" ) and Preprint Citation Index (Exclude – Database) | 3380315 |
|  | #3 | TS= (retraction of publication or retracted publication) and Preprint Citation Index (Exclude – Database) | 2257 |
|  | #4 | TS=(subgroup OR subpopulation or "post hoc" or post-hoc or substudy or subanalysis or subset or substrates or subtype or integrated) | 9508920 |
|  | #5 | #2 OR #3 OR #4 and Preprint Citation Index (Exclude – Database) | 12449724 |
|  | #6 | #1 AND #5 and Abstract or Meeting or Editorial Material or Letter (Exclude – Document Types) and News or Patent (Exclude – Document Types) | 3805 |
| **Cochran Library**  **5512** | #1 | ('Glucagon-Like Peptide-1Receptor' OR 'glucagon like peptide 1 receptor agonist' OR 'glp 1 agonist' OR 'glp 1 receptor agonist' OR 'glucagon like peptide 1 agonist' OR 'glucagon like peptide 1 receptor stimulating agent' OR 'long acting glp 1 agonist' OR 'long acting glp 1 receptor agonist' OR 'long acting glucagon like peptide 1 agonist' OR 'long acting glucagon like peptide 1 receptor agonist' OR 'dulaglutide' OR 'liraglutide' OR 'exenatide' OR 'albiglutide' OR 'semaglutide' OR 'lixisenatide' OR 'tirzepatide' OR ‘cotadutide’ OR ‘efpeglenatide’ OR ‘orforglipron’ OR ‘taspoglutide’ | 5753 |
|  | #2 | randomi?ed NEXT trial) OR "clinical trial" OR (randomi?ed) OR "random allocation" OR random* OR "controlled trial" OR "randomized controlled trial" | 1429796 |
|  | #3 | retraction of publication or retracted publication | 3867 |
|  | #4 | subgroup OR subpopulation or "post hoc" or post-hoc or substudy or subanalysis or subset or substrates or subtype or integrated | 127541 |
|  | #5 | #2 OR #3 OR #4 | 1448777 |
|  | #6 | #1 AND #5 | 5512 |
| **ClinicalTrials.gov**  **37** | #1 | 37 Studies found for: Studies With Results \| 'GLP-1 RA' OR 'glucagon like peptide 1 receptor agonist' Applied Filters: With Results | 37 |

**Table S2** Baseline characteristics of included studies

| **Trials** | **HbA1c (%)** | **Controls** | **Baseline  treatment** | **Indication for treatment** | **Interventions** |
| --- | --- | --- | --- | --- | --- |
| AWARD-1 | 8.1±1.3 | Placebo | Metformin + Pioglitazone | T2DM | Exenatide / Dulaglutide |
| AWARD-2 | 8.15 ±1.00 | Insulin glargine | Metformin +  Glimepiride | T2DM | Dulaglutide |
| AWARD-3 | 7.60 ±0.90 | Metformin | Lifestyle  intervention | T2DM | Dulaglutide |
| AWARD-4 | 8.43 ±1.05 | Insulin glargine | Metformin | T2DM | Dulaglutide |
| AWARD-5 | 8.15 ±1.10 | Sitagliptin/Placebo | Metformin | T2DM | Dulaglutide |
| AWARD-6 | 8.10 ±0.80 | / | Metformin | T2DM | Liraglutide /  Dulaglutide |
| AWARD-8 | 8.4 ±0.7 | Placebo | Glimepiride | T2DM | Dulaglutide |
| DURATION-1-6 | 8.5 ±1.1 | / | / | T2DM | Exenatide |
| DURATION-8 | 9.3 ±1.1 | Dapagliflozin | Metformin | T2DM | Exenatide |
| Jastreboff 2023 | 5.5 ± 0.4 | Placebo | Lifestyle  intervention | Obesity | Retatrutide |
| STEP-1^*^ | 5.7 ± 0.3 | Placebo | Lifestyle  intervention | Obesity | Semaglutide |
| SUSTAIN-6 | 8.7±1.45 | Placebo | / | T2DM | Semaglutide |
| SUSTAIN-7 | 8.23 ±0.90 | / | Metformin | T2DM | Dulaglutide / Semaglutide |
| SUSTAIN-China | 8.10 ±0.90 | Sitagliptin | Metformin | T2DM | Semaglutide |

**Notes:** HbA1c, glycated hemoglobin; T2DM, type 2 diabetes mellitus

^*^: The weight change from baseline in STEP-1 is evaluated using the percentage change in body weight; therefore, the unit is percentage rather than kilograms. This study was not included in the meta-analysis that used kilograms as the outcome measure.

**Table S3** Detailed characteristics of included studies

| **Trials** | **Sample  Size** | **Treatment  duration** | **Age (year)** | **Number of males** | **Number  of females** | **BMI (kg/m2)** | **Weight at baseline (kg)** | **Interventions** | **Weight reduction from baseline (kg)** |
| --- | --- | --- | --- | --- | --- | --- | --- | --- | --- |
| AWARD-1 | 276 | 26 weeks | 55±10 | 156 | 120 | 34±5 | 97±19 | Exenatide 10ug | Overall: -1.07±4.82 Male: -1.23±4.52 Female: -0.89±4.36 |
| AWARD-1 | 269 | 26 weeks | 56±9 | 164 | 105 | 33±6 | 96±21 | Dulaglutide 0.75mg | Overall: 0.2±4.76 Male: 0.15±4.48 Female: 0.25±4.26 |
| AWARD-1 | 271 | 26 weeks | 56±10 | 157 | 114 | 33±5 | 96±20 | Dulaglutide 1.5mg | Overall: -1.3±4.77 Male: -0.92±4.47 Female: -1.87±4.30 |
| AWARD-2 | 266 | 52 weeks | 57±9 | 133 | 133 | 32±5 | 86±18 | Dulaglutide 0.75mg | Overall: -1.33±3.91 Male: -1.14±3.12 Female: -1.71±3.12 |
| AWARD-2 | 263 | 52 weeks | 56±10 | 141 | 122 | 31±5 | 85±18 | Dulaglutide 1.5mg | Overall: -1.87±3.89 Male: -1.39±3.15 Female: -2.23±3.04 |
| AWARD-3 | 265 | 26 weeks | 56±11 | 117 | 148 | 33±6 | 92±19 | Dulaglutide 0.75mg | Overall: -1.36±3.91 Male: -0.91±3.59 Female: -1.75±3.69 |
| AWARD-3 | 265 | 26 weeks | 56±10 | 112 | 153 | 34±6 | 93±19 | Dulaglutide 1.5mg | Overall: -2.29±3.91 Male: -1.77±3.59 Female: -2.71±3.79 |
| AWARD-4 | 275 | 52 weeks | 59.3 ±9.0 | 138 | 137 | 33.1 ±5.2 | 91.7 ±18.0 | Dulaglutide 0.75mg | Overall: 0.18±4.48 Male: 0.61±4.05 Female: -0.33±4.21 |
| AWARD-4 | 273 | 52 weeks | 58.9 ±9.6 | 148 | 125 | 32.0 ±5.1 | 91.0 ±18.2 | Dulaglutide 1.5mg | Overall: -0.87±4.47 Male: -0.33±4.07 Female: -1.63±4.25 |
| AWARD-5 | 297 | 104 weeks | 54±10 | 130 | 167 | 31±4 | 86±18 | Dulaglutide 0.75mg | Overall: -2.39±4.48 Male: -1.97±3.11 Female: -3.2±3.23 |
| AWARD-5 | 301 | 104 weeks | 54±10 | 145 | 156 | 31±5 | 87±17 | Dulaglutide 1.5mg | Overall: -2.88±4.28 Male: -2.56±3.1 Female: -3.77±3.15 |
| AWARD-6 | 300 | 26 weeks | 56.8 ±9.9 | 149 | 151 | 33.6 ±5.2 | 94.4 ±19.0 | Liraglutide 1.8mg | Overall: -3.61±3.81 Male: -2.91±3.64 Female: -4.21±3.57 |
| AWARD-6 | 293 | 26 weeks | 56.5 ±9.3 | 135 | 158 | 33.5 ±5.1 | 93.8 ±18.2 | Dulaglutide 1.5mg | Overall: -2.9±3.8 Male: -2.35±3.59 Female: -3.28±3.59 |
| AWARD-8 | 236 | 24 weeks | 57.7 ±10.2 | 102 | 134 | 30.9 ±5.2 | 84.5 ±16.4 | Dulaglutide 1.5mg | Overall: -0.91±3.23 Male: -0.24±3.45 Female: -0.75±3.51 |
| DURATION-1-6 | 1719 | 24-30 weeks | 55±10.29 | 944 | 775 | 31.3±5.7 | 87.43 ±20.48 | Exenatide 2mg | Overall: -2.37±3.34 Male: -2.1±3.14 Female: -2.7±3.55 |
| DURATION-8 | 184 | 28 weeks | 54 ±10 | 96 | 88 | 32 ±5.9 | 89.8 ±20.2 | Exenatide 2mg | Overall: -1.55±4.01 Male: -0.56±3.92 Female: -2.63±3.85 |
| Jastreboff 2023 | 69 | 48 weeks | 50.6±13.3 | 36 | 33 | 37.5±5.9 | 106.4±19.8 | Retatrutide 1mg | Overall: -8.7±7.84 Male: -7.8±6.21 Female: -9.6±8.74 |
| Jastreboff 2023 | 33 | 48 weeks | 50.8±11.9 | 17 | 16 | 37.3±5.9 | 108.0±26.3 | Retatrutide 2/4mg | Overall: -16.3±9.09 Male: -13.8±6.22 Female: -18.8±10.04 |
| Jastreboff 2023 | 34 | 48 weeks | 46.8±14.1 | 18 | 16 | 37.4±4.7 | 107.0±21.3 | Retatrutide 4mg | Overall: -17.8±8.92 Male: -15.1±9.55 Female: -20.3±6.1 |
| Jastreboff 2023 | 35 | 48 weeks | 48.7±11.1 | 18 | 17 | 37.0±5.5 | 108.6±20.9 | Retatrutide 4/8mg | Overall: -23.9±8.9 Male: -19.8±8.65 Female: -28.5±7.1 |
| Jastreboff 2023 | 35 | 48 weeks | 46.1±13.5 | 18 | 17 | 37.4±6.0 | 106.5±21.6 | Retatrutide 2/8mg | Overall: -21.7±8.3 Male: -21.4±6.33 Female: -22.8±7.97 |
| Jastreboff 2023 | 62 | 48 weeks | 45.8±12.2 | 32 | 30 | 37.4±6.0 | 108.0±21.7 | Retatrutide 2/12mg | Overall: -24.2±9.64 Male: -21.9±9.29 Female: -26.6±9.37 |
| STEP-1^*^ | 228 | 68 weeks | 48±12 | 76 | 152 | 37.6 ± 7.0 | 105.6 ± 21.8 | Semaglutide 2.4mg | Overall: -17.3±9.3 Male: -14.5±8.0 Female: -18.7±9.6 |
| SUSTAIN-6 | 826 | 104 weeks | 64.6±7.3 | 495 | 331 | 32.7 ± 6.29 | 91.8±20.3 | Semaglutide 0.5mg | Overall: -3.6 ± 12.33 Male: -3.49±10.61 Female: -3.65±10.72 |
| SUSTAIN-6 | 822 | 104 weeks | 64.7±7.1 | 518 | 304 | 32.9 ± 6.18 | 92.9±21.1 | Semaglutide 1.0mg | Overall: -4.9 ±12.32 Male: -4.31±10.62 Female: -5.89±10.69 |
| SUSTAIN-7 | 299 | 40 weeks | 55±10.6 | 160 | 139 | 33.6±6.9 | 95.6 ±23.0 | Dulaglutide 0.75mg | Overall: -2.3 ± 4.67 Male: -2±9.35 Female: -2.5±9.32 |
| SUSTAIN-7 | 299 | 40 weeks | 56±10.6 | 171 | 128 | 33.1±6.6 | 93.4 ±21.8 | Dulaglutide 1.5mg | Overall: -3.0 ± 4.67 Male: -2.5±9.45 Female: -3.9±9.49 |
| SUSTAIN-7 | 301 | 40 weeks | 56±10.9 | 169 | 132 | 33.7±7.1 | 96.4 ±24.4 | Semaglutide 0.5mg | Overall: -4.6 ± 4.86 Male: -3.9±9.35 Female: -5.5±9.32 |
| SUSTAIN-7 | 300 | 40 weeks | 55±10.4 | 162 | 138 | 33.6±6.5 | 95.5 ±20.9 | Semaglutide 1.0mg | Overall: -6.5 ± 4.85 Male: -5.8±9.45 Female: -7.3±9.49 |
| SUSTAIN-China | 288 | 30 weeks | 53.0±11.4 | 160 | 128 | 28.2 ±5.0 | 77.6±16.4 | Semaglutide 0.5mg | Overall: -2.9±7.11 Male: -2.1±7.06 Female: -2.8±7.05 |
| SUSTAIN-China | 290 | 30 weeks | 53.0±10.6 | 154 | 136 | 27.9 ±5.0 | 76.1±16.3 | Semaglutide 1.0mg | Overall: -4.2±7.13 Male: -3.2±7.05 Female: -4.4±7.09 |

**Notes:** Data were shown as mean ± SD. SD, standard deviation; BMI, body mass index.

^*^: The weight change from baseline in STEP-1 is evaluated using the percentage change in body weight; therefore, the unit is percentage rather than kilograms. This study was not included in the meta-analysis that used kilograms as the outcome measure.

Figure S1 Risk of Bias version 2 (RoB v2) for assessment of clinical trial studies


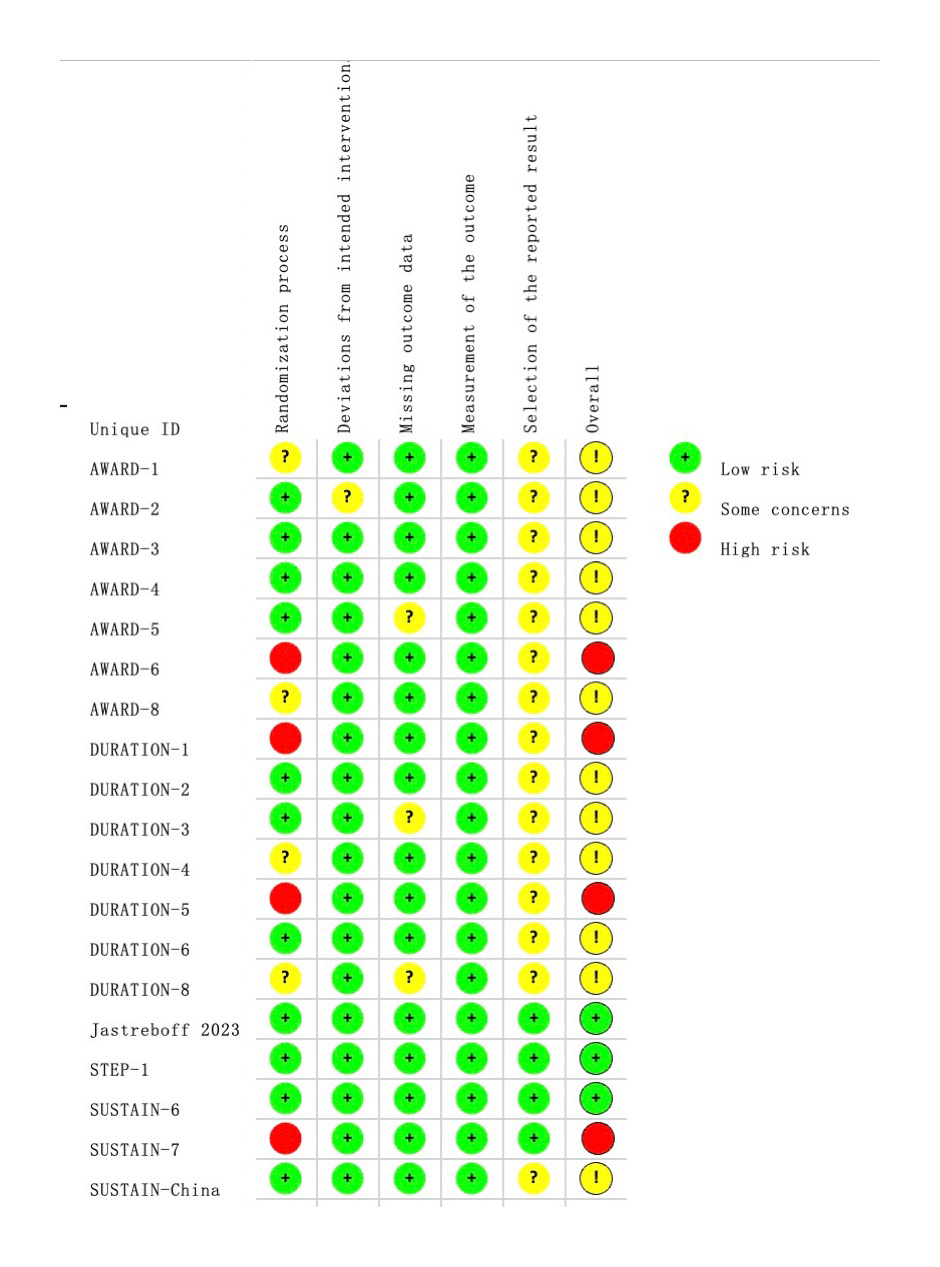


**Figure S2** Forest plot for meta-analysis of studies with weight reductions more than 5% of baseline body weight


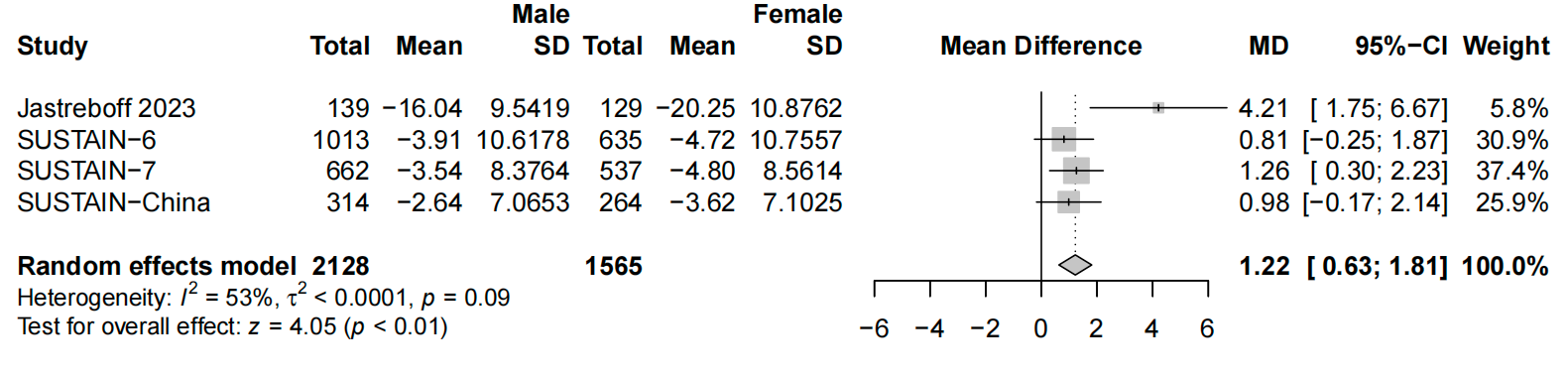


**Notes:** GLP-1 RA, glucagon-like peptide-1 receptor agonists; CI, confidential intervals

**Figure S3** Forest plot for meta-analysis of individual GLP-1 RAs


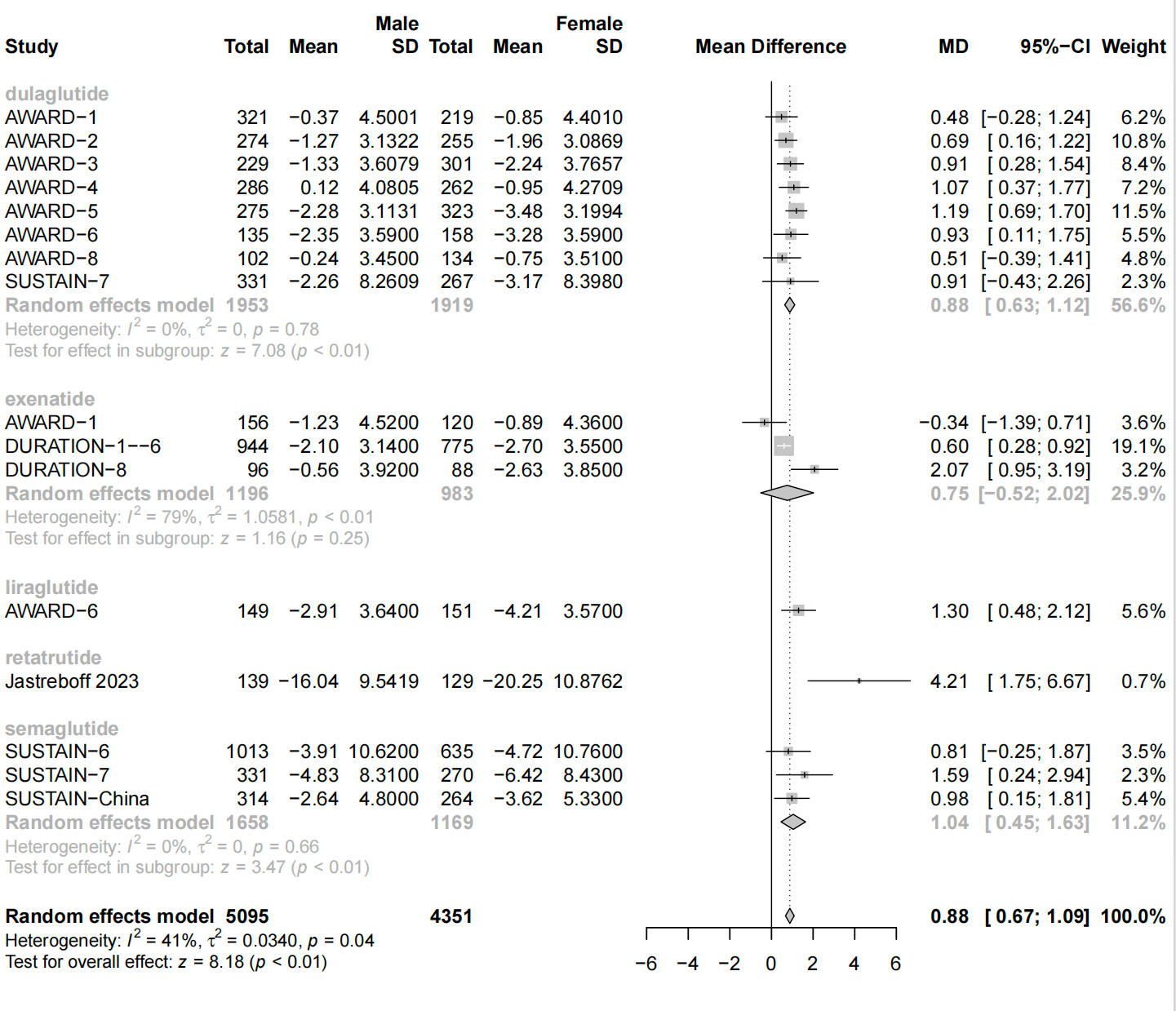


**Notes:** GLP-1 RA, glucagon-like peptide-1 receptor agonists; CI, confidential intervals

Figure S4 Effects of indication for treatment on the gender difference in weight reduction of GLP-1 RAs


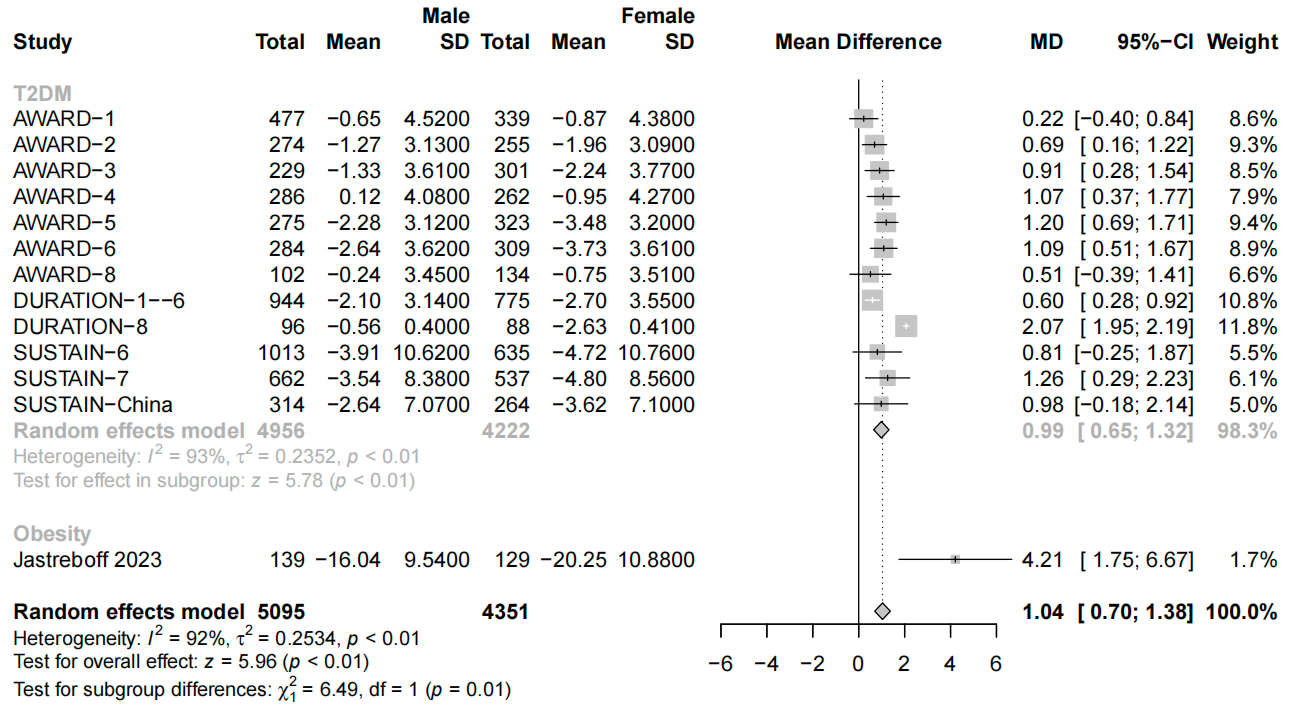


**Notes:** GLP-1 RA, glucagon-like peptide-1 receptor agonists; T2DM, type 2 diabetes mellitus; CI, confidential intervals

Figure S5 Effects of background treatment on the gender difference in weight reduction of GLP-1 RAs


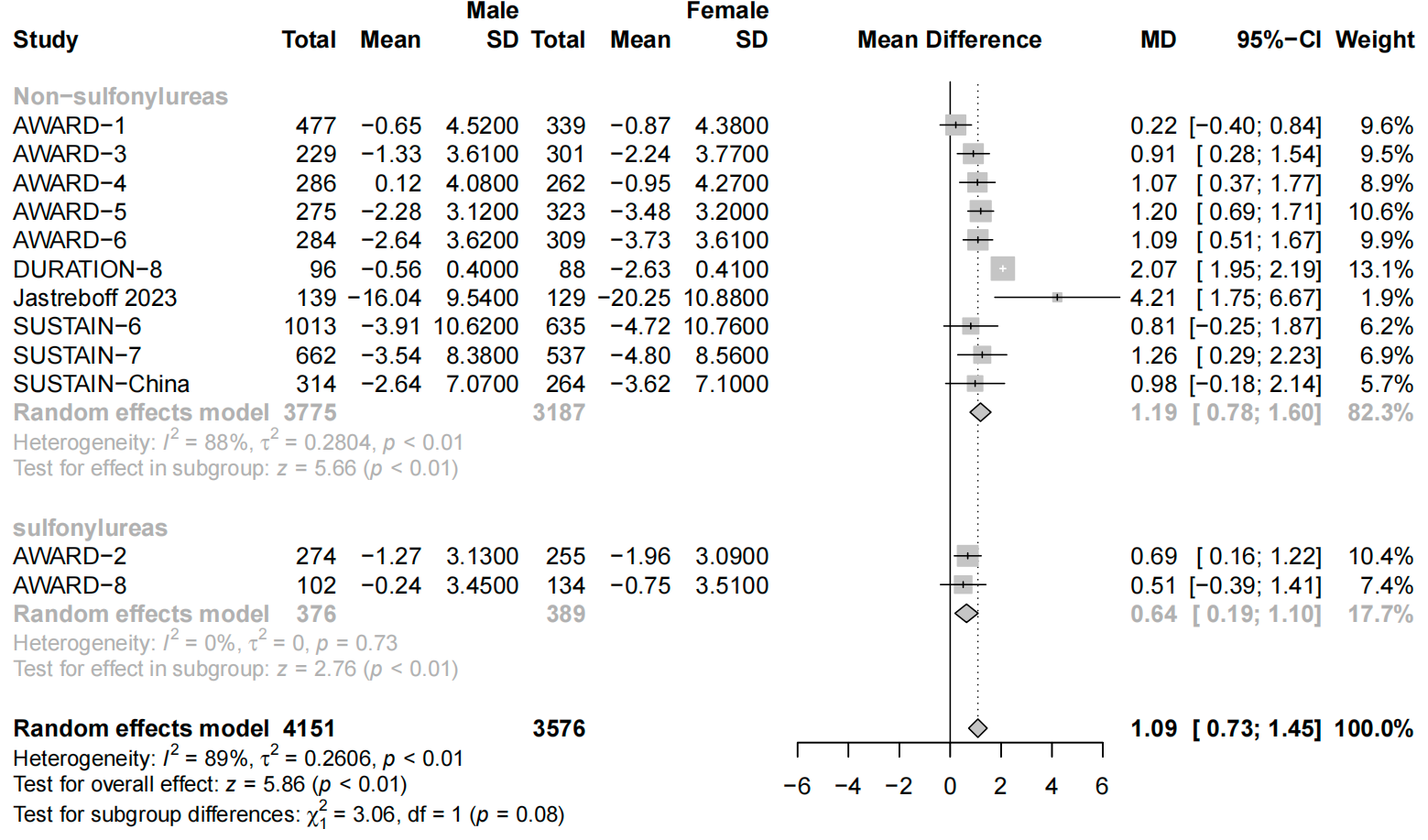


Notes: GLP-1 RA, glucagon-like peptide-1 receptor agonists; CI, confidential intervals

Figure S6 Effects of different doses on the gender difference in weight reduction of GLP-1 RAs


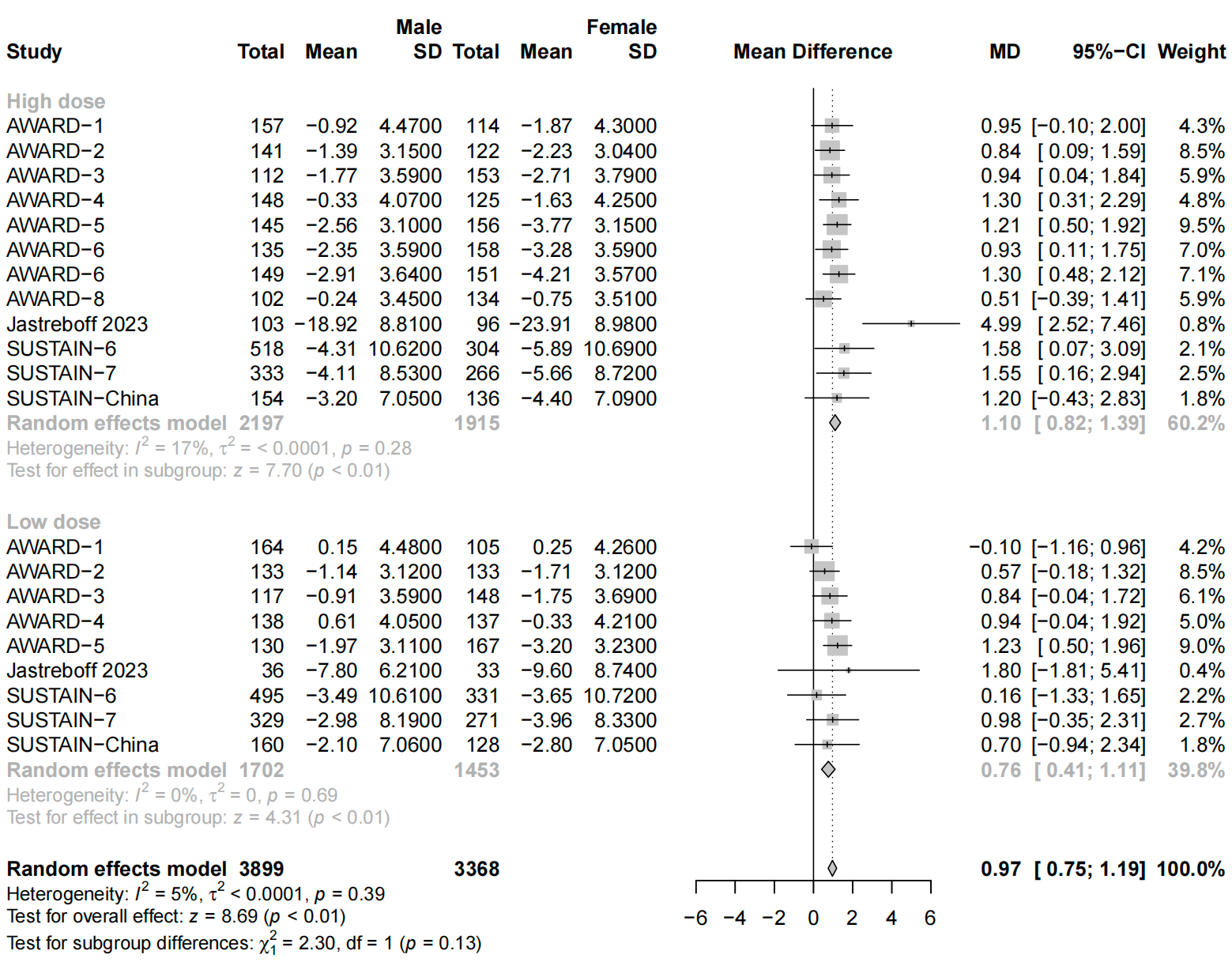


**Notes:** Among the studies included, only studies with dulaglutide, exenatide, subcutaneous semaglutide, and retatrutide had different doses of treatments. One trial might have two or more different dose of GLP-1RAs treatments. High dose of GLP-1RAs were defined as liraglutide ≥1.8mg once-daily, subcutaneous semaglutide ≥ 1.0mg once-weekly, dulaglutide ≥ 1.5mg once-weekly, retatrutide ≥ 12mg once-weekly; vice versa is the lower dose.

GLP-1 RA, glucagon-like peptide-1 receptor agonists; CI, confidential intervals

Figure S7 Effects of duration of treatment on the gender difference in weight reduction of GLP-1 RAs


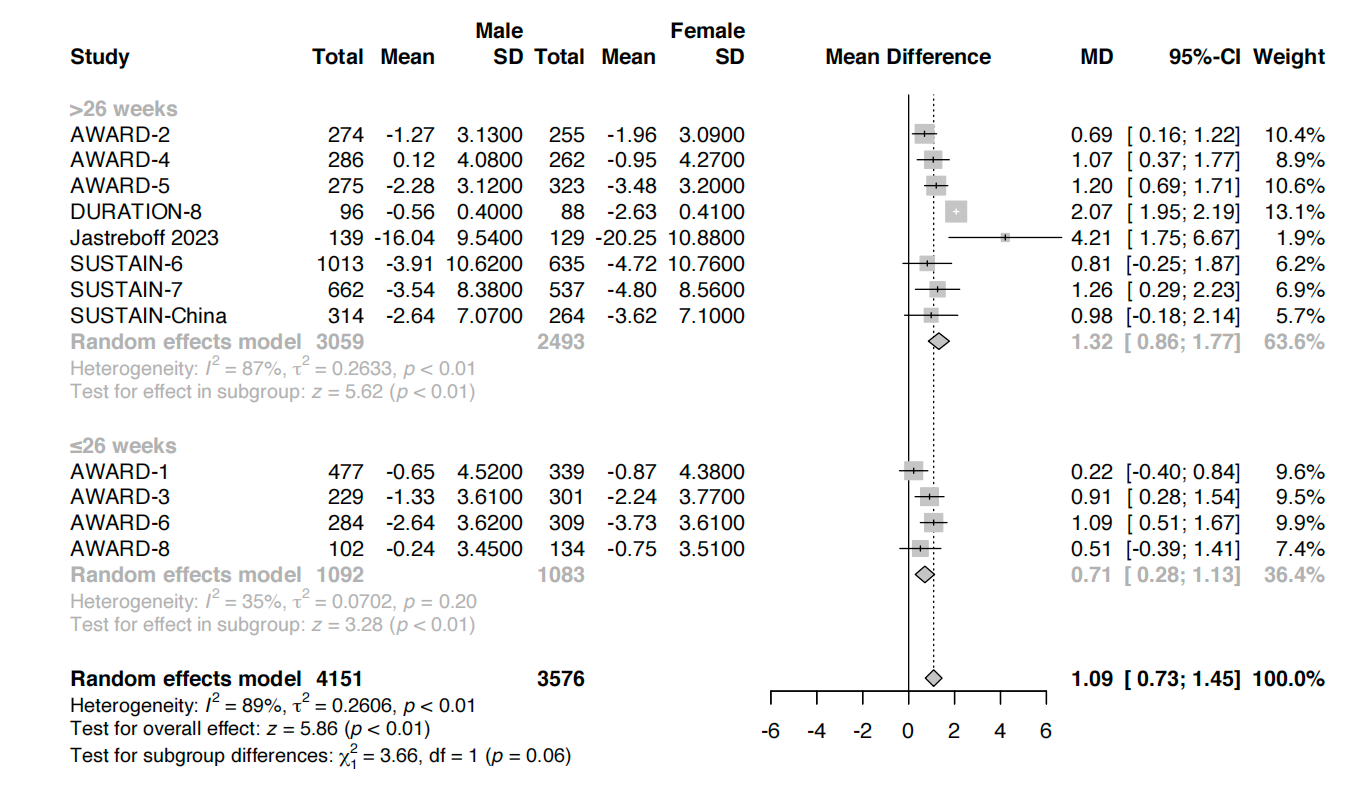


**Notes:** GLP-1 RA, glucagon-like peptide-1 receptor agonists; CI, confidential intervals

Figure S8 Effects of baseline body weight on the gender difference in weight reduction of GLP-1 RAs


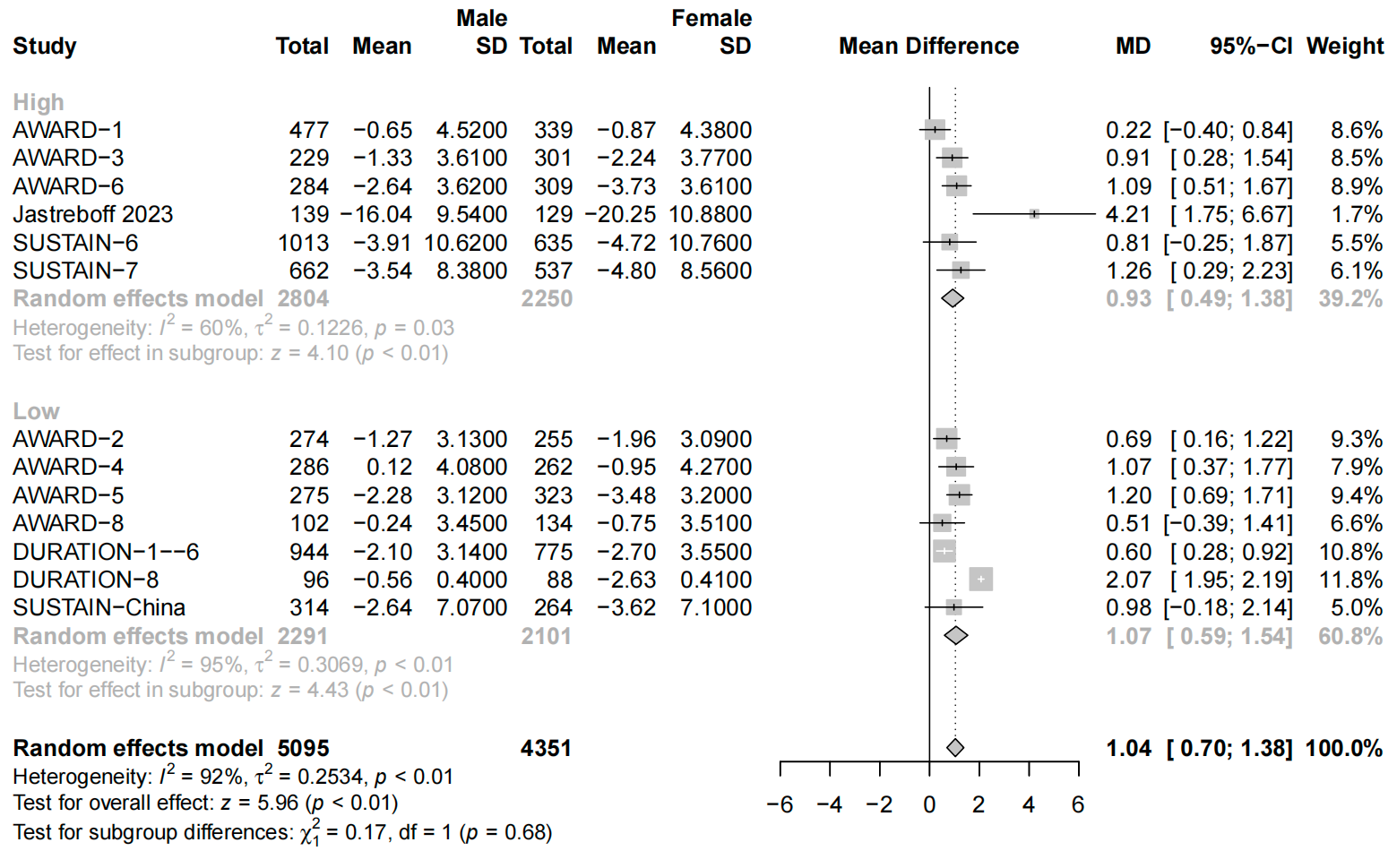


**Notes:** The cut-off point of the baseline body weight was 91.85kg which was the median of baseline body weight in all trials.

GLP-1 RA, glucagon-like peptide-1 receptor agonists; CI, confidential intervals

Figure S9 Effects of type of control on the gender difference in weight reduction of GLP-1 RAs


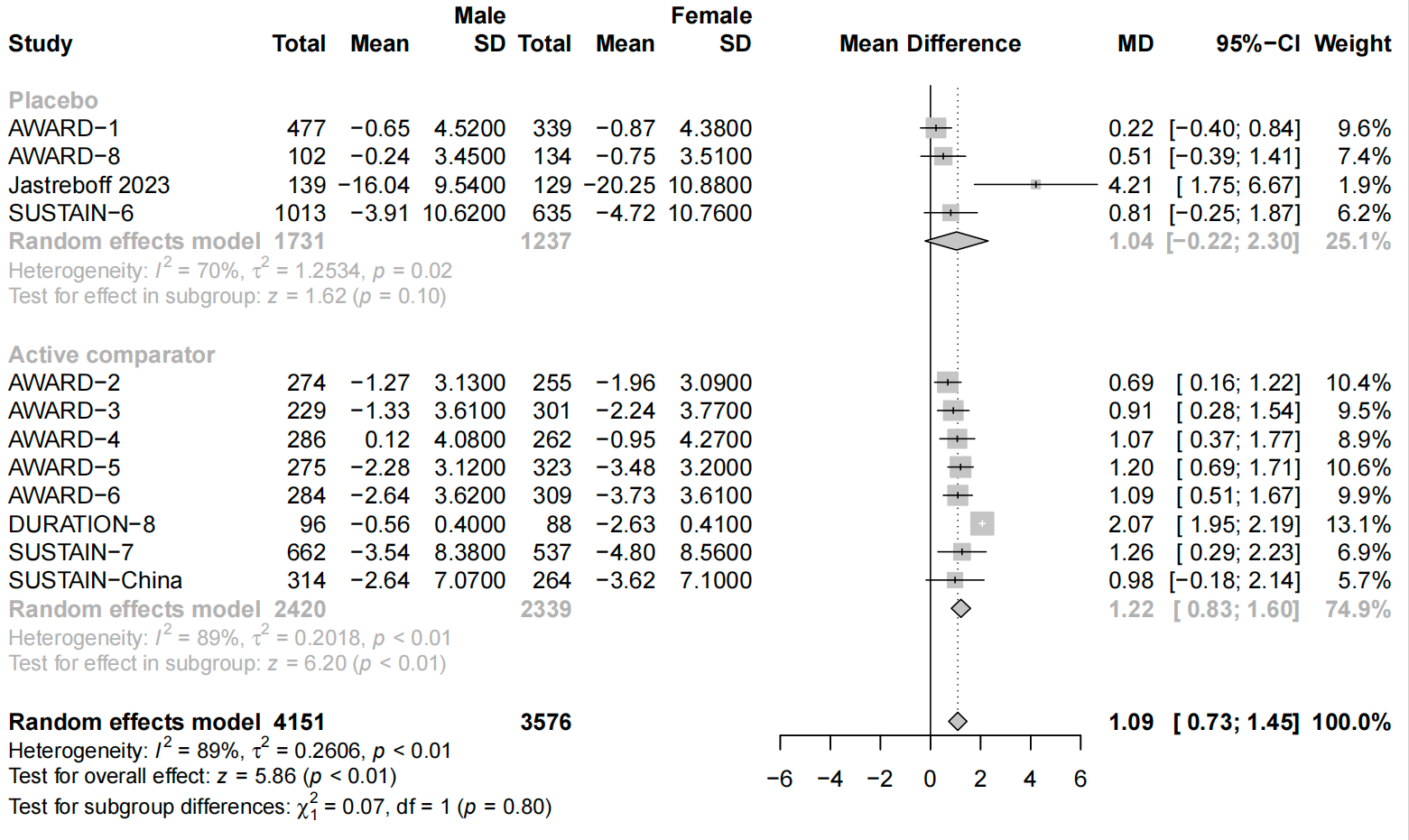


**Notes:** GLP-1 RA, glucagon-like peptide-1 receptor agonists; CI, confidential intervals

Figure S10 Effects of different doses on the gender difference in weight reduction of dulaglutide


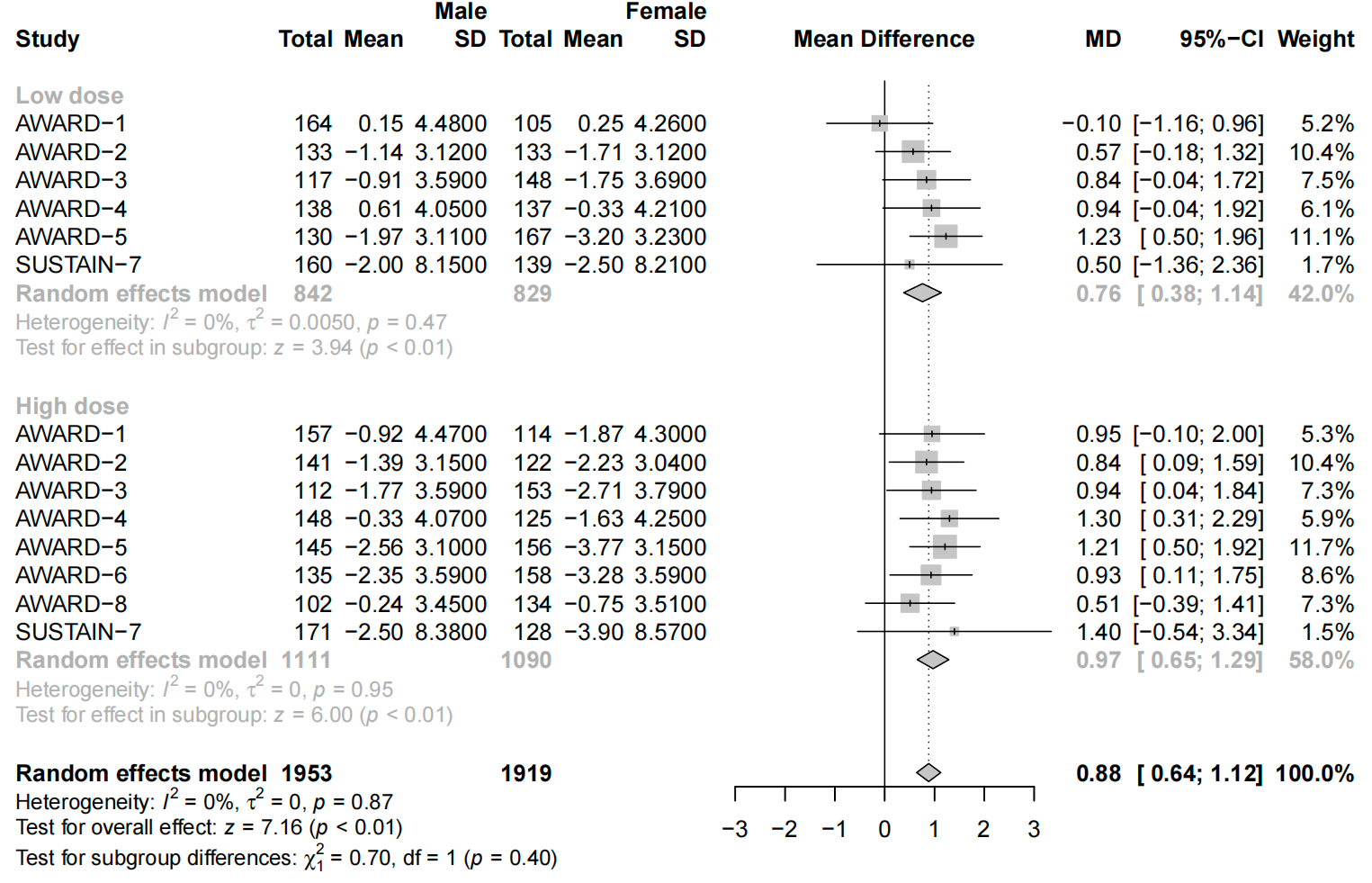


**Notes:** CI, confidential intervals

Figure S11 Effects of different doses on the gender difference in weight reduction of semaglutide


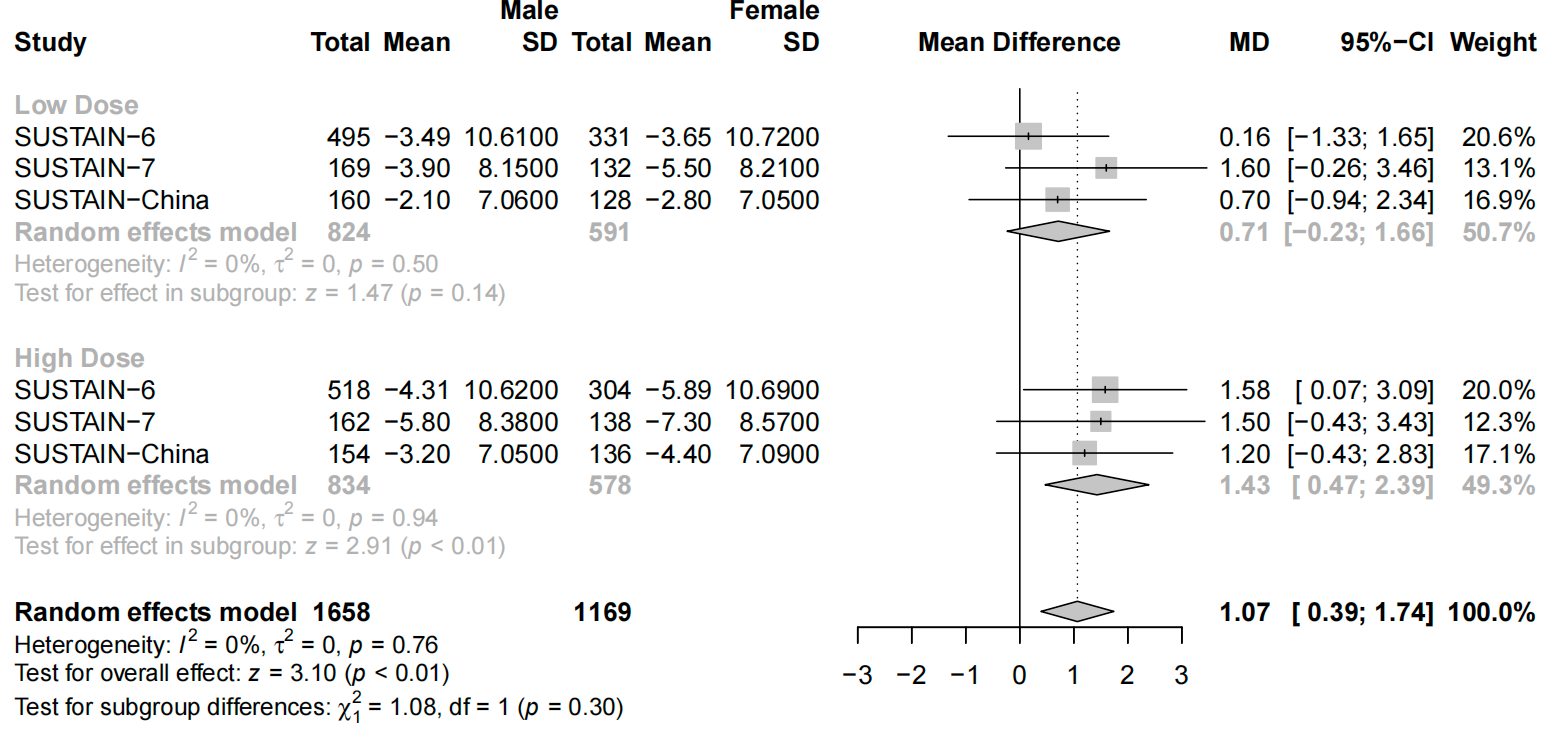


**Notes:** CI, confidential intervals

Figure S12 Effects of different doses on the gender difference in weight reduction of retatrutide


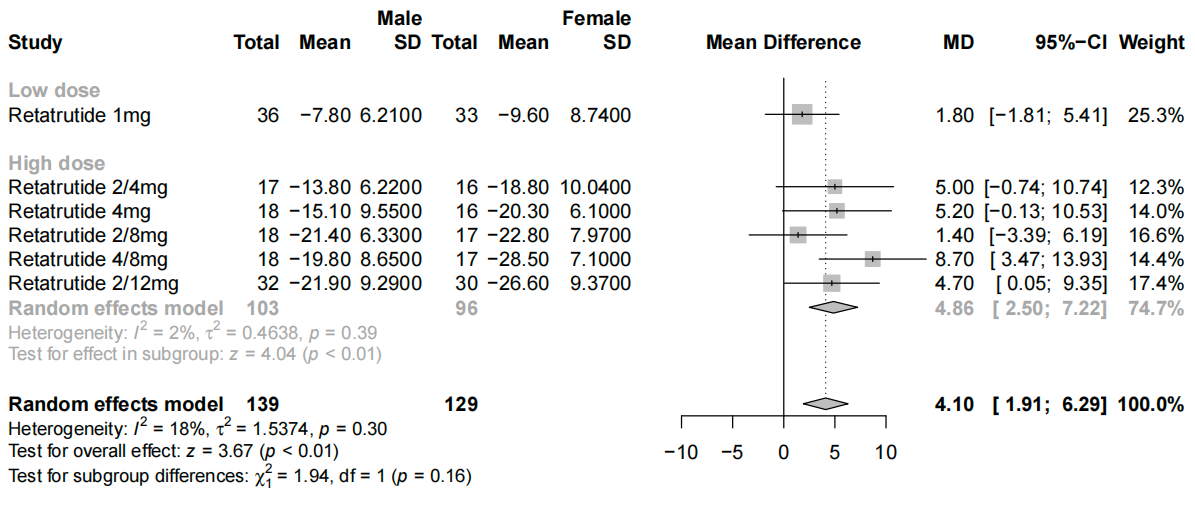


**Notes:** CI, confidential intervals

**Figure S13** Sensitivity analysis for pooled results of included studies excluding one with substantial weight reduction


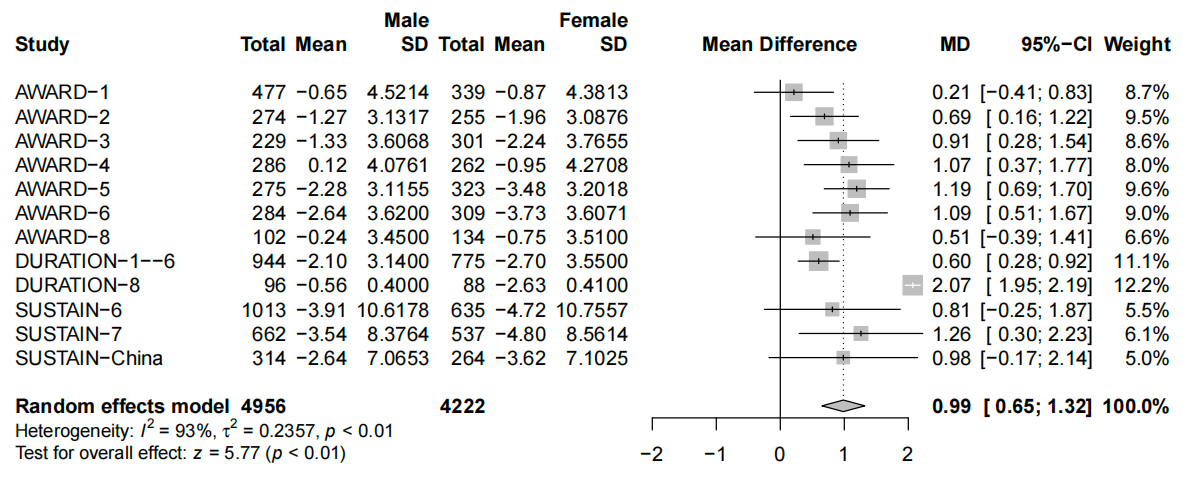


**Notes:** CI, confidential intervals

Figure S14 Sensitivity analysis for pooled results of GLP-1 RAs excluding retatrutide


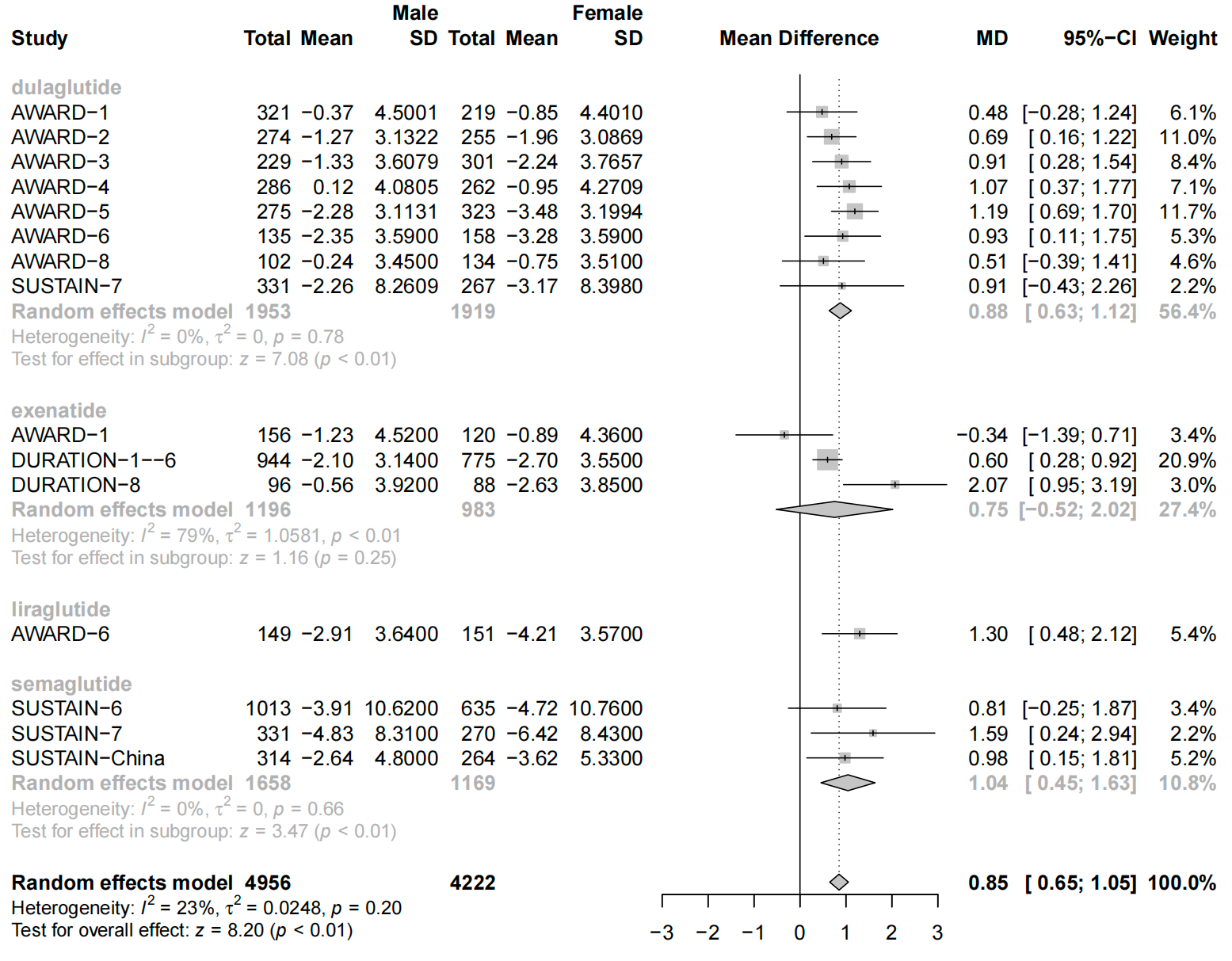


**Notes:** GLP-1 RA, glucagon-like peptide-1 receptor agonists

Figure S15 Funnel plot for meta-analysis results of included studies


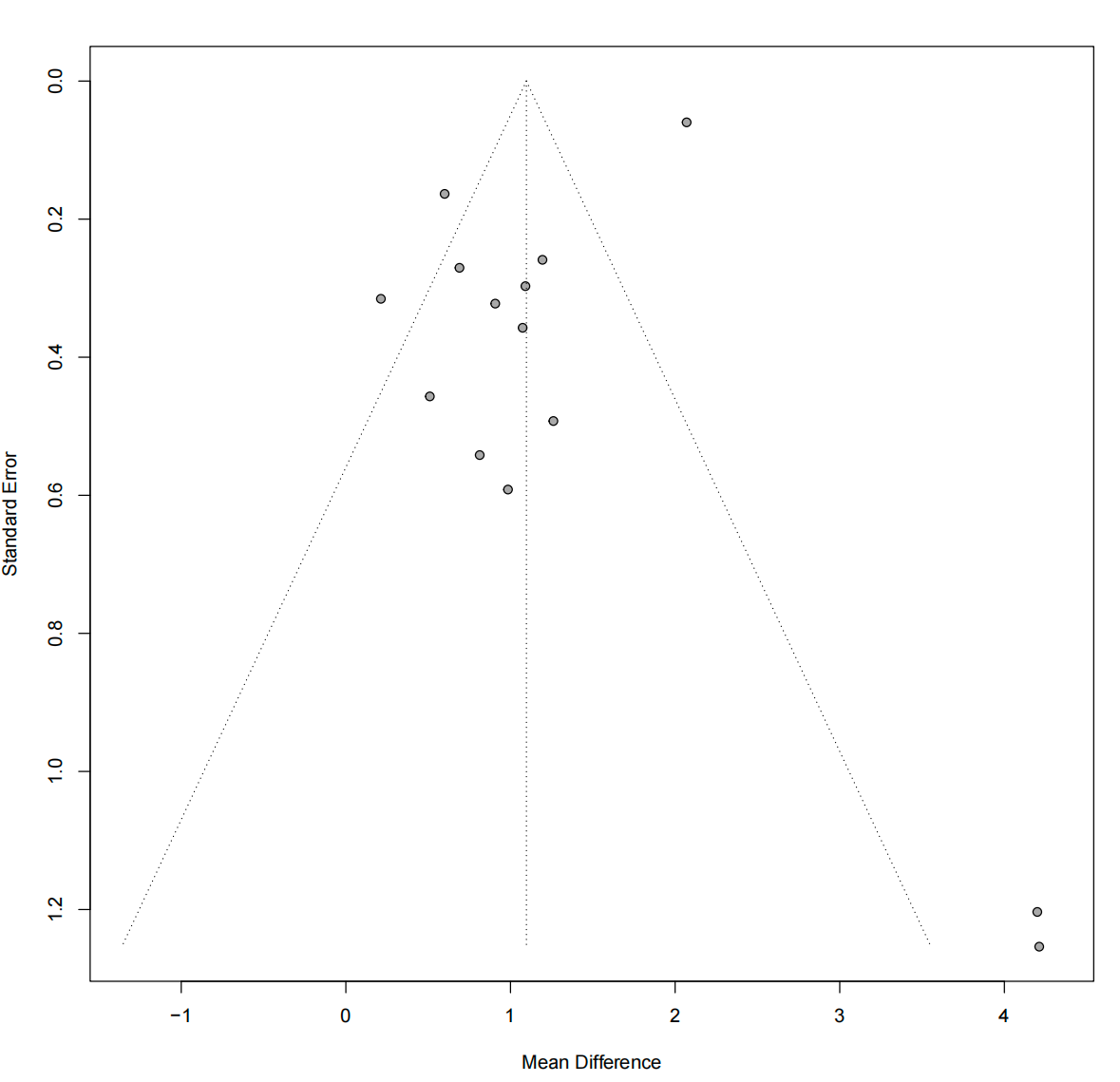

Supplement: Supplementary file 1 — Data S1. Supporting information. [file JDB-17-e70063-s001.docx]
